# Maintenance and Expansion: Modeling Material Stocks and Flows for Residential Buildings and Transportation Networks in the EU25

**DOI:** 10.1111/jiec.12216

**Published:** 2015-01-15

**Authors:** Dominik Wiedenhofer, Julia K. Steinberger, Nina Eisenmenger, Willi Haas

**Keywords:** construction and demolition waste, dynamic stocks and flows modeling, industrial ecology, material flow analysis (MFA), recycling, societal metabolism

## Abstract

Material stocks are an important part of the social metabolism. Owing to long service lifetimes of stocks, they not only shape resource flows during construction, but also during use, maintenance, and at the end of their useful lifetime. This makes them an important topic for sustainable development.

In this work, a model of stocks and flows for nonmetallic minerals in residential buildings, roads, and railways in the EU25, from 2004 to 2009 is presented. The changing material composition of the stock is modeled using a typology of 72 residential buildings, four road and two railway types, throughout the EU25. This allows for estimating the amounts of materials in in‐use stocks of residential buildings and transportation networks, as well as input and output flows. We compare the magnitude of material demands for expansion versus those for maintenance of existing stock. Then, recycling potentials are quantitatively explored by comparing the magnitude of estimated input, waste, and recycling flows from 2004 to 2009 and in a business‐as‐usual scenario for 2020. Thereby, we assess the potential impacts of the European Waste Framework Directive, which strives for a significant increase in recycling.

We find that in the EU25, consisting of highly industrialized countries, a large share of material inputs are directed at maintaining existing stocks. Proper management of existing transportation networks and residential buildings is therefore crucial for the future size of flows of nonmetallic minerals.

## Introduction

Ongoing efforts in the European Union (EU) to further improve its environmental performance and move toward a sustainable development path have led to the formulation of the Waste Framework Directive (WFD), which was put into national laws by 2010 (EPC 2008). Among pushes toward improved waste prevention and comprehensive national waste management plans, the directive also mandates quantitative goals for increased recycling as an important step toward a “recycling society”—especially construction and demolition waste, which is, after carbon emissions, the second largest waste stream of the European economies, has been targeted with a compulsory recycling rate of at least 70% of weight by 2020 (EPC 2008).

These steps have been taken while there are already large accumulated in‐use stocks of buildings and infrastructure throughout Europe. The central issue of stocks is that they usually have service lifetimes of at least years to decades, making them “suitable for depicting the influences a system's history has on its present—and hence for analysing temporal developments” (Faber et al. 2005, 155). Generally, stocks and the physical services they provide are closely linked to resource use and emissions, starting during extraction and processing of materials, during stock construction, while utilizing the services and operating the stock, for maintenance, and, finally, at the end of the service lifetime when demolished and landfilled or recycled (Pauliuk and Müller 2014). Material flow accounts for Europe consistently show that nonmetallic minerals make up 30% to 40% of domestic material consumption (Eurostat 2012a; Steger and Bleischwitz 2011; Steinberger et al. 2010; Weisz et al. 2006), all of which are accumulated as stocks. Aging buildings already require significant amounts of resource use for maintenance and refurbishment (Deilmann et al. 2009), especially when taking into account that their energy efficiency needs to be improved rapidly for effective climate‐change mitigation (Nemry et al. 2010; Meijer et al. 2009). But, at the same time, building stocks are still being expanded, thereby possibly running counter toward these efficiency efforts (Pauliuk et al. 2013a; Sandberg and Brattebø 2012). Linked with this expansion, especially of cities, is also the ongoing growth of already substantial transportation networks crisscrossing Europe (Steger and Bleischwitz 2011), inducing large maintenance requirements in materials, energy, and financial terms. At the same time, stocks can also be seen as a future secondary resource for urban or technospheric mining (Krook and Baas 2013; Brunner, 2011; Hashimoto et al. 2007). Therefore, systematic knowledge about the dynamics of in‐use stocks, their lifetimes, as well as expansion and maintenance requirements and subsequent end‐of‐life (EOL) waste flows and potentials for recycling are important stepping stones for a materially efficient (Allwood et al. 2011) recycling society and the potential transformation toward sustainability (Pauliuk and Müller 2014).

In this work, we utilize a dynamic material stocks and flows model for residential buildings and the road/railway network of the EU25 for 2004–2009 to investigate the relationships between these stocks and materials inputs and outputs of nonmetallic minerals. The changing material composition and size of stocks is modeled by utilizing detailed age/type matrices for 72 residential building types, four road and two railway types, combined with empirical growth and demolition rates as well as maintenance rates. In an otherwise business‐as‐usual scenario until 2020, some of the effects of strongly increased recycling mandated by the European WFD are explored.

## Literature Review and Scope Definition

The majority of existing literature on in‐use stocks and flows has thus far focused on metals (Müller et al. 2010; Graedel et al. 2013; Müller et al. 2014), with only a smaller proportion of studies investigating nonmetallic minerals, mostly from an industrial ecology and waste management perspective. Kapur and colleagues (2008, 2009) modeled the in‐use stocks of cement in the United States, using a top‐down dynamic model, thereby demonstrating that per capita stocks doubled over the past 50 years, and whereas in‐use stock increase is slowing down since the 1970s, they are still growing by approximately 2% to 3% per annum. Further, more than 80% of all cement utilized during the last century is still in use. Future maintenance and replacement requirements, especially for roads, are expected to be substantial because of stock aging (Kapur et al. 2009). Japanese studies further highlighted that, additionally to concrete, large amounts of sand, gravel, crushed stones, and aggregates are also stocked, with an overall in‐use stock increase of nonmetallic minerals by a factor of 3 between 1970 and 2000 (Hashimoto et al. 2009). Often, these materials are used in foundations and other more permanent infrastructure, which makes them unlikely to be recoverable, thereby substantially reducing the future potential recycling amounts (Hashimoto et al. 2009). Further, in Japan, stocked materials in various infrastructure, such as disaster prevention structures, harbors and airports, water and sewerage networks, and structures of the primary sector are each approximately the same amount as those in residential buildings and roads (Hashimoto et al. 2007). Studies on China highlighted the rapid accumulation of in‐use stocks of residential buildings (Huang et al. 2013), strong spatial inequality among urban and rural building stocks (Hu et al. 2010), as well as between Western hinterlands and coastal regions (Han and Xiang 2013) and explored potentials for dematerialization and a “circular economy,” also including the roads network (Wen and Li 2010; Shi et al. 2012; Guo et al. 2014).

A recent study on the cement cycle of Ireland, which also only recently turned into a booming economy, showed that EOL waste only amounts to 1% of concrete use because the majority of stocks have only been accumulated in the last 20 years (Woodward and Duffy 2011). One of the issues visible in this study is that the data quality on waste flows and collection rates is mixed, giving rise to substantial imbalances between EOL flows, recycling estimates, and reported waste (Woodward and Duffy 2011). Dynamic top‐down studies for Norway and the Netherlands, which are long‐standing wealthy economies, suggest that in‐use stocks of residential buildings are substantial and can be expected to keep growing slowly, with a probable saturation in the mid‐twenty‐first century, alongside strongly increasing EOL waste flows (Bergsdal et al. 2007; Müller 2006; Sartori et al. 2008) and subsequent challenges for waste management (Bohne et al. 2008). Overall, residential buildings in most of Europe are a mixture of different age and building types: 30% to 40% of buildings were built in the period 1945‑1970 and another 20% to 40% between 1971 and 1990 (Meijer et al. 2009; Nemry et al. 2010). Demolition rates have been ranging from 0.05% to 0.2% in the period 1980‑2005 (Thomsen and van der Flier 2009), resulting in a rapid accumulation of stocks across Europe. Maintenance, refurbishments, and replacements of these buildings to adapt to current and future standards, also in regard to climate‐change mitigation, have large implications financially as well as for materials and energy use and emissions (Nemry et al. 2010; Pauliuk et al. 2013a; Sandberg and Brattebø 2012; Thomsen and van der Flier 2009).

Additionally, road networks constitute a significantly sized stock of nonmetallic minerals, especially in Japan with its stricter building standards owing to frequent natural disasters (Hashimoto et al. 2007; Kapur et al. 2008; Schiller 2007; Tanikawa and Hashimoto 2009). For European roads and railways, data exist on kilometers (km) of network, but not on detailed age/type distributions, hindering the application of a dynamic cohort‐based modeling approach. But as a result of short service lifetimes, compared to buildings, material turnover can be expected to be much higher (Schiller 2007; Steger et al. 2011; Tanikawa and Hashimoto 2009).

In this study, the following research questions are posed, also with the aim of evaluating the European level data situation for future policy recommendations:

1. What is the size of in‐use stocks of nonmetallic minerals in residential buildings and the road/railway network of the EU25? What are the magnitudes of directly related flows of nonmetallic minerals into and out of these stocks resulting from maintenance and expansion? How do they compare to economy‐wide material use?
2. What are the dynamics of these stocks and flows over time and how could increased recycling under the WFD until 2020 affect these flows?

One of the major constraints for this study is one of data availability on stock characteristics, material composition and age distributions, as well as demolition and maintenance rates. Although it is clear that stocks in various infrastructure as well as public and commercial buildings are substantial, European‐level data sets are hardly available. Retrospective studies further face the problem of scale—although it is possible to exactly identify, for example, the material content of a single house or a road, doing so for a whole country is impossible. Different approaches exist to deal with this, for example, with representative building typologies (Nemry et al. 2008, 2010), average material intensities per floor space and building period (Bergsdal et al. 2007; Müller 2006; Schiller 2007), coupling of spatial databases of settlement structures, building standards and age distributions (Tanikawa and Hashimoto 2009), linking consumption of materials with service‐lifetimes and waste factors (Cochran and Townsend 2010), and combining input‐output methods with data on floor space, building regulations, and construction activity (Hashimoto et al. 2007).

## Methodology: Definitions, Data, and the Model

We apply a dynamic bottom‐up approach, utilizing time series of extents (*E*) of each stock type (*s*), either in number of buildings or km of road and rail, by country (*c*) and year (*t*), as well as material intensities (*M*), in metric tons, for each stock type (*s*) and material (*m*) (see supporting information available on the Journal's website for details of compilation; tables 1 and 2). These material intensities are grouped in “concrete and asphalt” as well as “other construction minerals” (bricks, stones, tiles, sand and gravel, and aggregates). Combined, these two groups constitute 96% of domestic material consumption (DMC) of nonmetallic minerals in the EU25 (Eurostat 2012b). The remaining 4% are “salt, fertilizer, and other products.”

**Table 1:** Material intensities and maintenance cycles for residential building types, roads and railways^a^

***Zone 1: Northern Europe***									
	***Single family houses***			***Multi‐family houses***			***High‐rise buildings***		
	***(11 types)***			***(11 types)***			***(3 types)***		
Number of buildings, EU25 (2003)	21,353,400			1,629,200			234,200		
***Material intensities [metric tons per building]***									
	***min***	***avg***	***max***	***min***	***avg***	***max***	***min***	***avg***	***max***
***Structurally used***									
Concrete	0	119	216	0	1,493	3,565	3,176	3,627	4,530
Other construction minerals	94	178	425	0	681	2,542	0	280	840
***Nonstructural use***									
Other construction minerals	25	42	65	154	313	549	424	778	1,355
***Renovation cycles (Nemry et al***. 2008) ***[years per building]***									
**Nonstructural use**									
Other construction minerals	25	26	30	25	26	28	27	28	28
***Zone 2: Central Europe***									
	***Single‐family houses***			***Multifamily houses***			***High‐rise buildings***		
	***(11 types)***			***(11 types)***			***(3 types)***		
Number of buildings, EU25 (2003)	35,084,100			2,243,320			129,440		
***Material intensities [metric tons per building]***									
	***min***	***avg***	***max***	***min***	***avg***	***max***	***min***	***avg***	***max***
***Structurally used***									
Concrete	0	119	184	0	1,257	3,402	2,312	2,936	4,185
Other construction minerals	0	143	384	0	732	2,547	0	456	1,368
***Non‐structural use***									
Other construction minerals	32	43	54	227	269	404	11	351	567
***Renovation cycles (Nemry et al***. 2008) ***[years per building]***									
***Non‐structural use***									
Other construction minerals	24	26	28	26	26	27	26	26	26
***Zone 3: Southern Europe***									
	***Single‐family houses***			***Multifamily houses***			***High‐rise buildings***		
	***(9 types)***			***(10 types)***			***(3 types)***		
Number of buildings, EU25 (2003)	2,412,500			296,600			3,100		
*Material intensities (metric tons per building)*									
	***min***	***avg***	***max***	***min***	***avg***	***max***	***min***	***avg***	***max***
***Structurally used***									
Concrete	0	142	211	0	1,198	3,402	2,312	2,936	4,185
Other construction minerals	0	89	384	0	400	2,547	0	912	1,368
***Non‐structural use***									
Other construction minerals	32	40	54	150	254	404	476	537	567
***Renovation cycles (Nemry et al***. 2008) ***[years per building]***									
***Non‐structural use***									
Other construction minerals	24	26	29	25	26	27	26	26	26

***EU road and railway network***						
	***Motorways***	***State roads***	***Provincial***	***Communal***	***Single track***	***Double tracks***
km in EU25 (2003)	58,310	383,944	1,380,274	3,336,382	125,132	74,764
***Material intensities (metric tons per km)***						
Asphalt, concrete	14,341	3,489	2,932	1,393	308	616
Other construction minerals	25,864	8,011	6,280	4,208	3,419	6,837
***Infrastructure lifetimes (sources in Supporting Information on the Web) (years per km)***						
Asphalt, concrete	17	20	20	20	47	47
Other construction minerals	60	60	60	60	60	60

a Structural use covers, for example, load‐bearing walls and foundations; non‐structural use includes roofs, bricks in non‐load bearing walls, tiles, plaster, etc; see the supporting information on the Web for details and compilation procedures.

Min = minimum; Avg = average; Max = maximum; EU = European Union; km = kilometers.

**Table 2:** Overview on major parameters of stock extent and change^a^

Composition of dwelling stocks and share of residential buildings not covered (Nemry et al. 2008, 18)					Housing growth and demolition rates			Average change of roads network 2000–2009				Average change of railways network 2000–2009		
				Not	Growth		Demolition							Recycling rate
	Single		High‐	represented by					State			Single	Double	of C&D
	family	Multifamily	rise	dwelling types	Avg 2003–09	2010+	Avg 2003–09	Motorways	roads	Provincial	Communal	tracks	tracks	waste
Austria	41%	46%	1%	12%	1.2%	1.2%	0.54%	0.4%	–0.2%	0.1%	0.0%	0.6%	1.4%	60%
Belgium	63%	20%	2%	15%	0.7%	0.7%	0.15%	0.4%	0.1%	0.1%	0.6%	–0.3%	0.6%	68%
Cyprus	50%	20%	0%	30%	4.0%	0.8%	0.03%	1.6%	0.7%	0.8%	2.5%	0.0%	0.0%	1%
Czech Republic	28%	30%	18%	24%	0.7%	0.7%	0.04%	2.7%	0.1%	0.0%	0.9%	0.3%	–0.1%	23%
Denmark	40%	33%	6%	21%	0.8%	0.8%	0.15%	2.7%	–2.0%	–0.3%	0.2%	–0.8%	0.7%	94%
Estonia	27%	32%	25%	16%	0.6%	0.6%	0.15%	2.2%	0.7%	1.0%	1.0%	1.4%	0.6%	92%
Finland	38%	47%	0%	15%	1.1%	1.1%	0.15%	4.9%	0.0%	0.0%	1.8%	0.0%	0.2%	26%
France	40%	28%	10%	22%	1.6%	1.6%	0.07%	2.2%	–5.0%	0.4%	0.9%	–0.7%%	0.2%	62%
Germany	41%	42%	4%	13%	0.5%	0.5%	0.12%	1.0%	–0.3%	0.1%	0.2%	–1.8%	0.3%	86%
Greece	44%	31%	0%	25%	0.8%	0.8%	0.15%	0.6%	–2.3%	0.0%	0.0%	0.3%	1.4%	5%
Hungary	42%	20%	14%	24%	0.8%	0.8%	0.11%	3.3%	0.1%	–0.1%	0.0%	0.3%	–0.1%	16%
Ireland	70%	4%	0%	26%	2.6%	0.8%	0.74%	2.3%	0.3%	0.7%	0.3%	–1.6%	–0.4%	80%
Italy%	34%	39%	12%	15%	0.8%	0.8%	0.15%	0.2%	0.5%	0.9%	0.7%	–0.6%	1.6%	0%
Latvia	24%	65%	0%	11%	0.8%	0.8%	0.10%	0.0%	–0.1%	0.1%	1.0%	–0.6%	0.2%	46%
Lithuania	31%	56%	0%	13%	0.2%	0.2%	0.01%	0.4%	0.1%	2.4%	3.5%	–0.4%	–1.3%	60%
Luxembourg	42%	17%	8%	33%	0.8%	0.8%	0.15%	0.4%	0.0%	0.0%	0.0%	0.0%	0.0%	46%
Malta	50%	30%	0%	20%	0.8%	0.8%	0.15%	0.0%	0.0%	4.3%	0.0%	0.0%	0.0%	0%
Netherlands	50%	28%	5%	17%	0.3%	0.3%	0.29%	1.3%	–0.3%	–0.1%	0.5%	–0.6%	0.6%	98%
Poland	35%	36%	18%	11%	0.5%	0.5%	0.04%	1.5%	0.3%	–0.1%	0.5%	–1.3%	–0.1%	28%
Portugal	44%	16%	14%	26%	1.0%	1.0%	0.15%	2.8%	2.1%	–0.8%	0.3%	–0.6%	2.2%	5%
Slovakia	43%	23%	16%	18%	0.3%	0.3%	0.02%	2.4%	0.9%	–0.4%	–0.2%	–0.1%	–0.1%	0%
Slovenia	47%	23%	8%	22%	0.7%	0.7%	0.04%	4.5%	1.7%	0.4%	0.3%	0.3%	0.0%	53%
Spain	26%	27%	22%	25%	8.5%	0.8%	0.20%	5.3%	–0.9%	–0.3%	0.1%	–0.2%	2.5%	14%
Sweden	40%	45%	0%	15%	0.4%	0.4%	0.03%	3.0%	0.4%	0.0%	0.4%	–0.2%	1.7%	0%
UK	53%	18%	1%	28%	0.9%	0.9%	0.08%	0.8%	–1.3%	1.0%	0.2%	0.6%	0.6%	65%
**Average EU25**	42%	31%	7%	20%	1.1%	0.8%	0.15%	1.9%	–0.2%	0.4%	0.6%	–0.2%	0.5%	47%

a See the Supporting Information on the Web for annual data as used in the modeling and details of compilation and detailed sources.

C&D = construction and demolition; Avg = average.

Using the MATLAB programming language, the following equation is used to estimate annual material stocks (*MS*) of nonmetallic minerals as sum over the multiplication of extents (*E*) by respective material intensities for each stock type (*S*), country (*c*), and year (*t*) (equation 1).

(1) $$MS_{c,m,t}=\sum_{s}E_{S,c,t}*M_{S,c,m}$$

For residential buildings and their material compositions, a typology of 72 “typical” buildings, developed by Nemry and colleagues (2008, 2010), representing 80% of residential buildings in the EU25 for the year 2003, is used (table 2; see the Supporting Information on the Web; Nemry et al. [2008]). Time series on annually finished and demolished dwellings and total dwelling stocks are compiled from Eurostat, European Housing Statistics reports ,and other national sources for 2003–2009 (see the Supporting Information on the Web for annual data and sources; table 2 for an overview). A recent survey, also using various sources for the 2000s and highlighting large data gaps showed that, in EU27 plus Norway and Switzerland, residential buildings constitute 75% of total European floor space, whereas the remaining square meters are commercial (15%), educational (4%), sport (1%), hospitals (2%), and of “other” (3%) nature (BPIE 2011). For these very heterogenous building types, no data on material composition are available. Data on roads and railways are compiled from Eurostat, UNECE, and the European Road Foundation, and material intensities for four road and two railway types were taken from the literature (see the Supporting Information on the Web; table 1). Because of very mixed data quality, various data points had to be either linearily interpolated or taken from the EU‐wide average (see the Supporting Information on the Web for detailed documentation). No similar data on other infrastructure, such as bridges, tunnels, dams, ports, sewers, and so on, were available.

Based on these time series, we also constructed the business‐as‐usual scenario for 2020, using the average national growth and demolition rates from 2003 to 09 (table 2) as well as the lifetimes for each stock type that are subject to maintenance (see the Supporting Information on the Web; table 1). For this scenario, the banking and subsequent public debt crisis of 2007–2008 is also taken into account: For countries that experienced stronger‐than‐average 2% growth of their dwelling stock between 2003 and 2009 (Spain, France, and Ireland), the average of the remaining EU25 countries (0.78%) was used for the projection from 2010 onward.

Expansion of the stock of buildings and transportation networks and subsequent material inputs (*MI_expansion*) are defined by net increase of stock extent and are calculated in two steps: First, the annual net increase of stock extent^1^ (*E_add*) of stock type (*s*), in country (*c*) between year (*t*) and t+1, is calculated from equation (2) for *E_change* > 0, whereas the net decrease of extent (*E_decl*) uses equation (2), for *E_change* < 0.

(2) $$E\_change_{s,c,t+1}=E_{s,c,t+1}-E_{s,c,t}$$

This additional stock extent (*E_add*) is multiplied by the respective material intensity (*M*) for stype type (s), country (*c*), and material (*m*) (equation 3) to arrive at the material inputs due to expansion.

(3) $$MI\_expansion_{c,m,t}=\sum_{s}E\_add_{s,c,t}*M_{S,c,m}$$

Maintenance, on the other hand, is defined as material inputs required to keep the stock extent and the services it provides constant, despite stock aging and EOL flows. Maintenance therefore includes two material inputs: first, ongoing renovation cycles of nonstructural components of residential buildings, such as roofs, tiles, nonload carrying walls (table 1), as well as the renewal of worn down layers of a roads; second, also replacement constructions of stocks that were demolished because they were at the end of their service lives are included, for example, rebuilding of railway sections or roads, as well as buildings. The lifetimes for each building type from Nemry and colleagues (2008) are used to assign probabilities of demolition (i.e., the older a building, the higher the probability). For this short period of estimations and because of lack of data on the change of composition of residential buildings, we assume that each stock type (i.e., single‐family house and communal road) is replaced by a similar type, but of up‐to‐date construction standards. For roads and railways, we assume, based on the literature, that maintenance, in the form of renewal of the asphalt layers, happens every 17 to 20 years (depending on road type; table 1; see the Supporting Information on the Web), and for railways, the aggregates and concrete sleepers need replacement every 47 to 60 years (table 1; see the Supporting Information on the Web).

Maintenance material inputs (*MI_maintenance*) are therefore calculated in two steps: First, all roads, rails, and buildings (E) that are at the end of service life (*E_EoL*) and need replacement are calculated using country (*c*) and time (*t*) specific empirical demolition rates (*Demol*) for entire buildings and the reciprocal of lifetimes as an approximation of replacement requirements for roads and railways (*Demol*). Renovation cycles for nonstructural building components as well as upper road layers and railways sections are calculated by the reciprocal of the lifetimes (*LT*) from table 1 (equation 4):

(4) $$E\_EoL_{s,c,m,t}=E_{S,c,t}*Demol_{S,c,t}+E_{S,c,t}*\frac{1}{LT_{S,c,m}}$$

The maintenance material inputs (*MI_maintenance*) are then the sum over the multiplication of all EOL stocks (*E_EoL*) by the specific material intensity (*M)* of the new infrastructure/building type replacing it,^2^ as well as the material intensity (*M*) for the stock components that were renovated (Table 1 and supporting information available on the Journal's website) (equation 5).

(5) $$MI\_maintenance_{c,m,t}=\sum_{S}E\_EoL_{s,c,t}*M_{s,c,m}$$

The material outputs from stock (*MO*) are estimated by the EOL stocks *(E_EoL)* from equation (4) and the stocks coming out of use as a result of stock decline (E_decl), taken from equation (2). The sum over both stocks multiplied by the material intensity (*M*) of these specific EOL stocks then yields the total material output from stocks by country (*c*), material (*m*), and each year (*t*) (equation 6).

(6) $$MO_{c,m,t}=\sum_{S}\left(E\_EoL_{s,c,t}+E\_decl_{s,c,t}\right)*M_{s,c,m}$$

The amounts of recycled materials (*MR*) are calculated using country (*c*) specific recycling rates (*Recy_rate*) (equation 7). The remainder of the material output from stocks is then counted as construction and demolition waste, again not considering stock hibernation.

(7) $$MR_{c,m,t}=MO_{c,m,t}*Recy\_rate_{c}$$

These recycling rates were sourced from a recent metastudy for the European Commission (EC), which also highlighted the difficult data situation in regard to EOL waste flows and recycling estimates (Monier et al. 2011; table 2).

## Results on Material Stocks and Flows for the EU25

**Figure 1:**
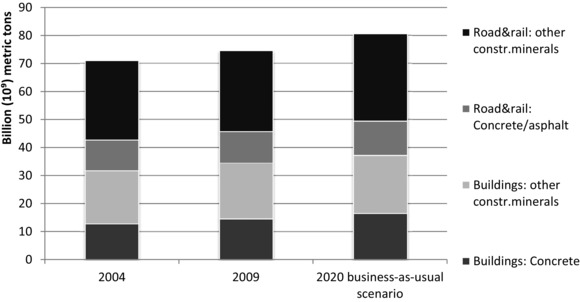
Stocks of nonmetallic minerals in residential buildings, roads, and railways in the EU25 (own calculations).

In‐use stocks of nonmetallic minerals in roads are estimated at 39 billion tons, in railways at 1 billion tons, and for residential buildings at 35 billion tons in 2009 (figure 1). On average, per capita stocks therefore amount to 128 tons of nonmetallic minerals in roads, 72 tons in residential buildings, and 3 tons in railways.

Between 2004 and 2009, stocks were expanded, with an average annual net increase of 160 million tons stocked in roads, 3 million tons in railways, and 542 million tons in residential buildings (figure 1). For the business‐as‐usual scenario until 2020, the increase of material stocks slows down to 0.7% per annum, which is a result of the reduction of housing growth rates in Spain, Ireland, and France (table 2; *Methodology* section). Still, total estimated stock increases to 81 billion tons of nonmetallic minerals in 2020 in that scenario.

**Figure 2:**
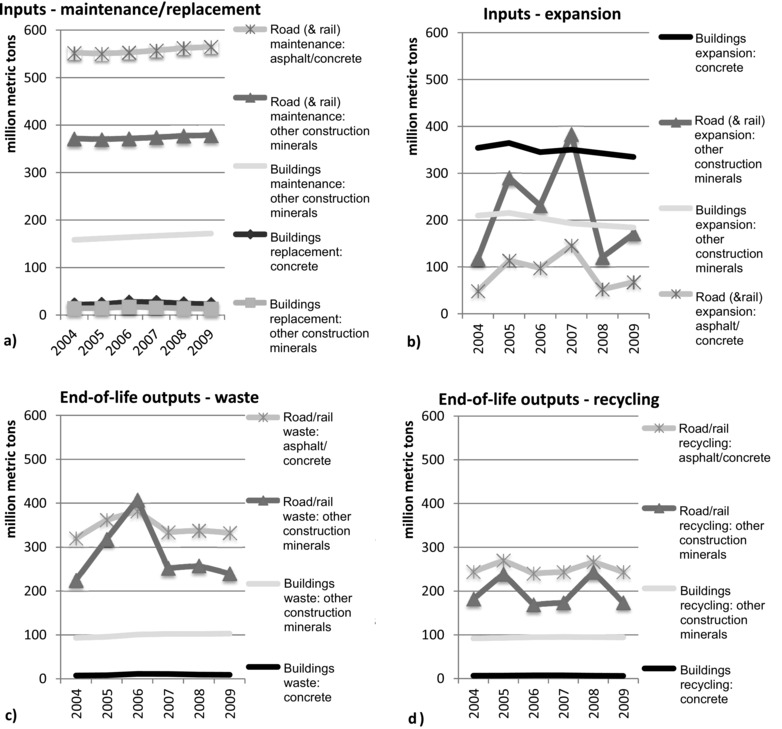
Material inputs and outputs from stocks. Strong fluctuations in road‐ and railway‐related flows, are to some extent, the result of mixed data quality (own calculations; see the Supporting Information on the Web).

Modeled inputs into stocks amount to 1,989 million tons, of which annual maintenance of the roads network makes up the majority, with on average of 47% or 930 million tons per annum. Maintenance inputs into buildings amount to 204 million tons, which are 10% of total estimated inputs, on average, between 2004 and 2009 (figure 2a,b). Replacement construction only takes up a small share of total inputs— on average, annually 24 million tons of concrete inputs and 15 million tons of other nonmetallic minerals. Expansion of the housing stock used 28% or 548 million tons of inputs annually, on average, whereas the remaining 15% or 307 million tons are related to the expansion of roads and, to a minor share, railways. Further, the modeled annual buildings expansion flows decreased slightly by approximately 3% between 2004 and 2009, reflecting the effects of the banking/public debt crisis and subsequent economic recession from 2007 onward.

Overall, modeled outputs from stocks amount to, on average, 1,178 million tons, of which construction and demolitions waste from roads makes up the largest fraction, with, on average, 49% or 628 million tons annually (figure 2c,d). Of that, outputs from railway stocks make up only 23 million tons, or 2%, on average. Buildings demolition waste amounts to 110 million tons or 8% of total output from stocks, on average. Overall, recycled construction minerals amount to approximately 550 million tons, of which 81%, on average, results from maintenance and demolitions of the roads and railway network, whereas the remainder stems from buildings stocks.

**Figure 3:**
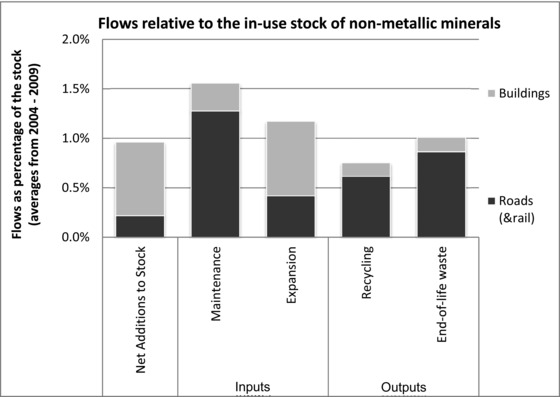
Modeled material flows and the quantitative relationship to the stock for 2004–2009.

The mass of estimated material flows into and from stocks are all in the range of only 0.8% to 1.6% of stocks (figure 3). The major share of net additions to stocks are the result of the expansion of the residential building stock (0.7%). In maintenance inputs, mostly roads and only lightly railways make up the majority (1.3%). For the outputs from stocks, materials from roads make up most of the recycling as well as waste.

### Bottom‐up Flows and Economy‐wide Material Consumption in 2009

**Figure 4:**
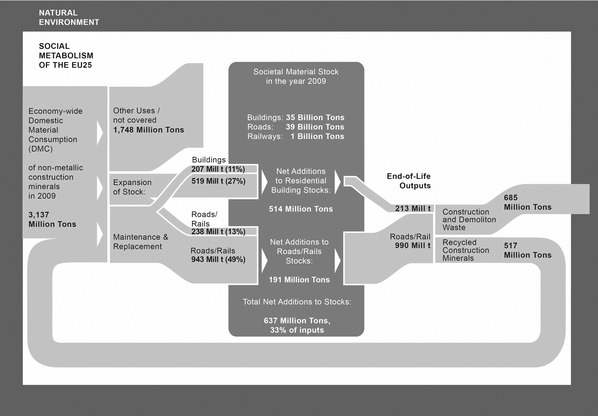
Economy‐wide consumption of nonmetallic minerals versus estimated inputs and outputs from stocks of residential buidlings, roads, and railways in 2009 (own calculations; DMC of non‐metallic minerals without “other products, salt and fertilizer”; Eurostat [2012a]). DMC = domestic material consumption.

Bottom‐up estimates of inputs into stocks can be compared with economy‐wide consumption figures. Inputs flows into stocks (above) are modeled at 1,908 million tons in 2009, which would account for 61% of overall DMC of construction minerals in the EU25 (figure 4). Over the whole time period 2004–2009, it is 56%, on average. But not all of those inputs are virgin materials—one has to consider that the recycling flows are, to some extent, already replacing those. This means that if all the estimated recycled materials are used to replace inputs into stocks and are not used for other purposes not covered in this study, still 1,389 million tons of virgin materials, or 44% of the DMC, is required for stock maintenance and expansion.

These results raise the question of where the “remaining” 39% to 56% of DMC of construction minerals used annually are destined. Uncertainties in model parameters (lifetimes, material intensities, demolition, and recycling rates) definitely play a role here. Additionally, these estimates do not cover all societal material stocks, for example, bridges, ports, airports, tunnels, underground networks, and commercial and public buildings are not included.

## Increased Recycling Resulting from the European Waste Framework Directive in an Otherwise Business‐as‐Usual Scenario for 2020

The European WFD (2008/98/EC) states, in Article 11, that “by 2020, the preparing for re‐use, recycling and other material recovery, including backfilling operations using waste to substitute other materials, of non‐hazardous construction and demolition waste […] shall be increased to a minimum of 70% by weight,” from the European average of 46% in 2004–2009 (Monier et al. 2011; Mudgal et al. 2011). Using the data and model described above (tables 1 and 2; *Methodology* section; Supporting Information on the Web), a business‐as‐usual scenario is modeled, in which only recycling rates are increased according to the EU WFD targets, whereas all other factors, such as demolition and growth rates^3^ as well as renovation and maintenance rates, are held constant (a so‐called ceteris paribus assumption).

**Figure 5:**
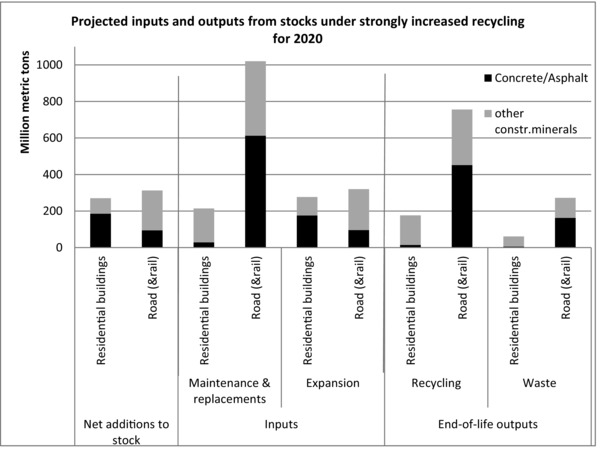
Projected results for the business‐as‐usual scenario exploring the effects of increased recycling under the Waste Framework Directive's goals in 2020.

In such a business‐as‐usual scenario, annual inputs of nonmetallic minerals into residential buildings and the road/railway network in 2020 are estimated at 1,829 million tons, which is a decrease of –4% compared to 2009 (figure 5). This decrease stems from reductions in expansion‐related inputs (–22% in 2020 compared to 2009), the majority of which is a result of reduced housing expansion in the scenario (–46% reduction of housing expansion material inputs). Maintenance inputs increase in the scenario (8% from 2009) and make up the majority of the modeled inputs in 2020 (55%), where all the concrete and 8% of other construction minerals go into replacement construction. Modeled outputs from stocks amount to 1,264 million tons, an increase of 5% compared to 2009. Increased recycling rates in 2020 result in 932 million tons of recycled materials, an increase of 70% over 2009. This means that if the WFD is fully implemented until 2020, 51% of virgin inputs into the stocks of residential buildings, roads, and railways could be sourced from recycled materials, given the otherwise business‐as‐usual scenario of ongoing stock expansion (figure 5). If all recycled materials are used for maintenance alone, 75% of these flows could be covered, materials quality considerations put aside.

### Verification of Results and Discussion of Limitations and Uncertainties

This study focuses on the European level and relies on a variety of European data sources, assumptions, and simplifications, all of which are outlined in the *Methodology* section and discussed in more detail in the Supporting Information on the Web. Major problems were low data quality on extent and change of dwelling stocks, which are collected at irregular intervals and published in the “Housing Statistics Reports.” For roads, data gaps and statistical breaks are common (see the Supporting Information on the Web). Research and policy recommendations would greatly benefit from improved data gathering and harmonization.

The few existing estimates of construction minerals stocks in selected EU countries have arrived at 130 to 325 tons per capita, lower estimates covering only residential buildings and upper estimates including all infrastructure, buildings, and subsurface constructions (Bergsdal et al. 2007; Daxbeck et al. 2009; Müller 2006; Rubli et al. 2005). Estimates in this study cover residential buildings and roads/rails and arrive at 203 tons per capita in 2009 for the EU average, of which housing accounts for 36% (72 tons). This comparatively low value for building stocks can be partly attributed to an underestimation resulting from incomplete primary data: The typology of 72 buildings that was used represents only 80% of the EU residential dwelling stock (Nemry et al. 2008, table 2).

Further, stocks in road infrastructure also seem underestimated, where a study for Germany arrived at 6.2 to 8.2 billion (10^9^) tons of stocks in the road system for 2009 (Steger 2012), whereas this study finds 5.5 billion tons in 2009, which is 12% to 32% lower. Germany‐specific material intensities, which are slightly higher than those used in this study, are the cause. Generally, the uncertainties for road and rail estimates are higher than for buildings because of the lack of information on the age composition of roads, the need for more specific material intensities over time and by region, as well as the overall low data quality for communal and provincial roads, which actually constitute the majority of the network (see the Supporting Information on the Web for Details).

In regard to stock growth, a recent study presented direct estimates of the economy‐wide “net additions to stock” for the Czech Republic, arriving at 58 million tons of net additions of construction minerals in 2002 (Kovanda et al. 2007). Estimates presented herein for 2005, although focused on the EU25 aggregate, amount to only 5 million tons for the Czech Republic. Although a direct comparison of the two values is complicated by the fact that the results refer to different years and that additions to stocks can vary considerably from year to year, it is clear that this study does underestimate net additions as a result of the different scope. Several factors contribute here: (1) This study only covers 76% of residential buildings of the Czech Republic (table 2), 92) roads and railways are estimated without any supporting infrastructure (bridges, tunnels, train stations, and so on), and (3) this study does not cover various other infrastructure and public or commercial buildings, whereas Kovanda and colleagues (2007) use an economy‐wide approach.

Recycling estimates of construction and demolition waste presented in this study agree well with a recent report to the European Environmental Agency (Tojo and Fischer 2011), which arrived at 554 million tons in 2007 for the EU27 versus the 488 million tons from this study for the EU25. As a waste estimate, this model yielded 685 million tons in 2007, which is 24% less than the 896 million tons reported by Tojo and Fischer (2011). Monier and colleagues (2011, 15) further conclude, in a review of the literature, that 310 to 700 million tons of construction and demolition waste for the EU27 is a plausible range. Eurostat recently included a first estimate of construction and demolition waste for the EU25 for 2010 in their database, which is 330 million tons (Eurostat 2013). Uncertainties for lifetimes and maintenance rates, material intensities, and demolition and recycling rates have to be mentioned here as issues. Further, the explicit calculation of road maintenance as flows, which involves in situ recycling, which usually only counts as waste if moved from the site in other studies, does affect the results. Additionally, “hibernating” stocks no longer in use, but not turned into a waste flow, as well as the ratio of below‐ and above‐ground stocks can play an important role in explaining these differences, given that they strongly influence the amounts of EOL stocks actually turning into waste flows (Hashimoto et al. 2009). Overall, the results of this study for waste and recycling are higher than the Eurostat numbers, but well within the ranges discussed by other European studies.

Summarizing, the results obtained from our model are lower than what more detailed country‐level studies find, although the overall size of comparable stocks is quite similar and waste and recycling estimates are in a very plausible range; the reasons for the underestimation in this study can be attributed to incomplete data at the EU scale, generally conservative assumptions, and no explicit treatment of stock hibernation, for which no data exist. Inclusion of commercial and public buildings, as well as other infrastructure, such as underground networks, sewers, ports, dams, and so on, would also definitely refine the results and substantially increase total stock estimates, as has been demonstrated for specific countries (Rubli et al. 2005; Hashimoto et al. 2007, 2009; Steger et al. 2011). An economy‐wide approach therefore would constitute an important further step.

## Outlook and Remaining Research Gaps

The majority of residential buildings in the EU25 have been constructed between 1945 and 1990 and their service lives are expected to end during the mid‐twenty‐first century, requiring strongly increasing replacement construction, recycling, and waste treatment (Bergsdal et al. 2008; Müller 2006). Based on past housing survival, these projections assume 60 to 120 years of service life. But empirical demolition rates for buildings are generally very low, with a EU25 average of 0.15% (table 2, 0.01–0.5%; Thomsen and van der Flier [2009]). Implicitly, this would be a lifetime for the entire stock of 667 years (inverse of demolition rate), which is not a realistic figure, but shows that this projected strong increase of demolitions is not yet happening. Because demolitions are usually driven by other factors than actual technical EOL (Thomsen and van der Flier 2011), lifetime extension and increased focus on renovations and refurbishments are an important policy option toward more sustainable and efficient resource use. In this way, the material input as well as the waste and recycling side could be tackled while also making the building stock more energy efficient (Pauliuk et al. 2013a). Additionally, also roads, but probably also other infrastructure, have to be included in considerations toward more sustainable resource use, given that they constitute a large part of the material stock and also induce large material flows (Hashimoto et al. 2009; Schiller 2007; Steger 2012).

At this stage of research, no explicit recycling loops or cascading uses are included, rather a comparison of the magnitudes of material flows is discussed. As a next step, one would need to consider first the temporal and spatial distribution of stocks and flows resulting from the severe economic limitations on transporting and storing large quantities of recycled construction and demolition flows. Second, specific material qualities and their quantities would need to be included to gain an understanding of the actual feasibility for recycling and usability. This goes beyond this study and current data availability and is a topic for further research.

Further, an inverse relationship between buildings density and material stocked in roads and other infrastructure has been reported (Schiller 2007; Pauliuk et al. 2013b), indicating the importance of spatial planning and reducing urban sprawl. Careful densification of settlements is also being advocated for climate mitigation strategies in order to reduce the energy requirements of transportation (Newman and Kenworthy 1999; Wiedenhofer et al. 2013 and references herein). In combination, this could lead to reduced traffic loads, prolonging the lifetimes of road infrastructure and decreasing their large maintenance requirements and monetary burden on state budgets. This indicates that substantial cobenefits toward more sustainable material use and climate strategies might exist, which have to be understood more clearly (Deilmann 2009; Weisz and Steinberger 2010; Allwood et al. 2011).

## Conclusions

We presented a dynamic model of material stocks in residential buildings and the road/railway network and the material flows going into their maintenance, as well as net expansion, for the EU25. Results are on the conservative lower side, compared to other studies, with per capita stocks estimates for nonmetallic minerals at 128 tons in roads, 72 tons in residential buildings, and 3 tons in railways in 2009 for the EU25.

Interestingly, maintenance‐related material inputs into the stocks covered in this study amount to 34% to 58% of domestic material consumption of nonmetallic minerals in the EU25 in 2009, depending on how recycling is handled. The majority of these flows are the result of maintenance of roads, then for the renovation of buildings. Additional net expansion of stocks amounts to approximately another 28% of DMC of nonmetallic minerals, of which the majority is used for additional residential buildings. Overall, the results indicate a significant commitment of annual resource use for maintaining existing stocks.

In 2020, strongly increased recycling in an otherwise business‐as‐usual scenario has been estimated to only cover 51% of material input flows into residential buildings and roads/rails resulting from ongoing stock expansion. But, if all recycled materials are used for stock maintenance alone, 75% of these flows could be covered. This scenario is based on trend extrapolations from 2003 to 2009 and an increase of recycling rates for construction and demolition waste from the European average of 47% during that time toward the WFD's goal of a minimum of 70% in 2020.

Based on the results presented in this article and in line with other studies (Hashimoto et al. 2007; Müller 2006; Shi et al. 2012), the following insights emerge: The size of stocks as well as their service lifetimes are the two most important factors driving material use necessary for renewal and maintenance. This, in turn, means that a reduction of material use would be most easily achieved through a stabilization of existing stocks (“steady stocks”) and an effort to prolong lifetimes of standing infrastructure and buildings. Preliminary results for European roads suggest that these stocks are a major driver of resource use, and their maintenance and net expansion need to be considered critically.

Finally, serious efforts toward more sustainable patterns of society‐nature interactions need much more systematic considerations on how to use the substantial material stocks already in use in industrialized countries more efficiently and much longer. The majority of these structures have been accumulated during the rapid acceleration of the fossil‐fuel–based system of high resource throughput (Fischer‐Kowalski 2007; Pauliuk and Müller 2014), when sustainability was not an issue. Moving toward resource‐saving longer lifetimes, improved maintenance, and renovation practices, additionally to an overall stabilization or sometimes even shrinking of material stocks and flows, therefore amounts to a critical paradigm shift (Allwood et al. 2011; Boulding 1966).

## Supporting information


**Supporting Information S1:** This supporting information contains the procedures and data sources used to compile the data sets on the residential buildings stock and road/railway network of the EU25 states.

## Notes

1 Only in few countries and specific years was a decline of infrastructure extent found and then only mostly for single‐track railway lines and minor roads. These are deducted from the time series E and turn into end‐of‐life waste.

2 End‐of‐life buildings are replaced by a building type of current standards, that is, a single‐family house from 1960 is replaced by a single‐family house type of current building standards and designs.

3 Housing growth rates for Spain, Ireland, and France have been reduced to the remaining EU average for 2010 onward, to take the economic recession into account (see *Methodology* section).
