# Supplementary material for: Maintenance and Expansion: Modeling Material Stocks and Flows for Residential Buildings and Transportation Networks in the EU25
Source: J Ind Ecol. 2015 Jan 15;19(4):538–51. doi: 10.1111/jiec.12216 (PMC4965784; doi:10.1111/jiec.12216)
Supplement: Supplementary file 1 — Supporting Information S1: This supporting information contains the procedures and data sources used to compile the data sets on the residential buildings stock and road/railway network of the EU25 states. [file JIEC-19-538-s001.pdf]

SUPPORTING INFORMATION FOR:

Wiedenhofer, D., J.K. Steinberger, N. Eisenmenger, and W. Haas. 2014. Maintenance and expansion: Modeling material stocks and flows for residential buildings and transportation networks in the EU25. *Journal of Industrial Ecology.*

Summary

This supporting information contains the procedures and data sources used to compile the datasets on the residential buildings stock and road/railway network of the EU25 states.

Summary

This supporting information contains the detailed documentation on the data sources and procedures used to compile a consistent dataset on the extent of the residential housing stocks and the road and rail infrastructure of the EU25 member states. Furthermore parameters on service lifetimes, material intensities, demolition and growth rates as well as recycling rates used in the main article are discussed and their compilation is documented.

The primary goal of this work is to gain first estimates on standing stocks of construction minerals, related material flows and recycling potentials, especially in the light of current EU level efforts towards a ‘reycling society’. Although country level studies could offer more detail in data, parameters and assumptions, the foucs of this work is on the European overall perspective. This means that generally for this work preference was given to EU level statistics like Eurostat and EU level publications like the EU Housing reports. Were necessary additional statistical databases as well as literature sources were used.

Due to data constraints this dataset only covers residential buildings, roads and railways. Not included are all other commercial and public buildings (malls, factories, airports, government buildings, schools, etc), all sorts of additional infrastructure like bridges, tunnels, parking lots as well as supporting infrastructures and other public works like dams, sewers, and undergrounds.

Content

Materials covered in this modelling exercise 3

Residential buildings: Data sources and compilation procedures 4

The 72 residential housing types 6

Estimating growth and demolition rates for the EU25 housing stocks 14

Growth of the dwelling stock 15

Road and rail network: Data sources and compilation procedures 19

Infrastructure extent and temporal dynamics 19

Material composition of road and railway infrastructure 21

Service lifetimes of the road and railway infrastructure 22

Literature 23

Appendix A: Time series data of road and rail infrastructure extent for all EU25 members from 1990-2009 25

Materials covered in this modelling exercise

Due to the scope of this study, the sub-groups ‘chemical and fertilizer minerals’ (MF3.4), ‘salt’ (MF3.5), ‘other n.e.c.’ (MF 3.9.) have been excluded.

Table S1: Summary of materials covered in the modelling and the appropriate material flows analysis (MFA) material types

**Non-metallic construction minerals**	**Concrete**
**Materials covered in the housing data**	
Clay	Aerated concrete element, density 0,6 reinforced
Clinker	
Exterior plaster, lime-cement scratch	Concrete c20/25
Gravel	Concrete roof tiles
Gypsum boards	Light-weight concrete, pumice hollow block
Interior plaster, lime-gypsum	
Limestone	Light-weight concrete, expanded clay block
Roof tiles	
Rubble stone masonry, density 1,6	Ready-mix concrete, c20/25
Sand	Reinforced concrete
Sand-lime bricks	
Screed, anhydrite	
Screed, cement	
Vertically perforated bricks	
**Materials covered in the road/railway data**	
Concrete	Gravel & sand
Asphalt	Other non-metallic filling materials, crushed stones, etc
Bitumen	

**MFA Category 3. "Non-metallic minerals"**	**Shares in DMC of non-metallic Minerals, 2004**	**Covered in the model**
**MFA data**		
3. Non-Metallic Minerals	100%	95.5%
3.1 Marble, granite, sandstone, porphyry, basalt, other ornamental or building stone (excluding slate)	5.8%	Yes
3.2 Chalk and dolomite	2.0%	Yes
3.3 Slate	0.1%	Yes
3.4 Chemical and fertilizer minerals	1.1%	Yes
3.5 Salt	1.7%	Excluded
3.6 Limestone and gypsum	18.1%	Yes
3.7 Clays and kaolin	3.1%	Yes
3.8 Sand and gravel	64.1%	Yes
3.9 Other n.e.c.	3.5%	Excluded
3.10 Excavated earthen materials (including soil), only if used (optional reporting)	0%	Excluded

Residential buildings: Data sources and compilation procedures

To estimate the dynamics of the residential building stock we build on a large number of data and literature sources. Firstly we use published work from (Nemry et al., 2010, 2008), who presented a typology of 72 detailed residential houses, representing 80% of the EU25 residential housing stock for the year 2003. Secondly the report on 'Housing Statistics in the European Union 2010' (Statistics Netherlands, 2012) and other national statistical sources were used to compile parameters on country-level growth and demolition rates of the residential housing stock. Thirdly both data sources had to be brought together for the purpose of this study – the procedures and assumptions applied are also documented and discussed below.

Using these sources and procedures (all described in detail below), the dynamics of the residential dwelling stock of the EU25 is estimated to behave in the following way, where the time period from 2003-2009 is based on real data and the 2010-2020 period is based on trend extrapolations (Figure S1, upper left; see below for detailed discussions). The effects of the financial crisis from 2007 onwards are clearly visible, where the annual rates of increase of the stock slow down markedly, from around 1.7% p.a. to 0.8% p.a. (Figure S1; upper right). This is mostly due to the strong decrease of national level stock increase rates, where all countries with growth rates larger than 2% (Spain, Ireland, France) have been reduced to the average of the remaining EU25 countries. This clearly translates into a strong decrease of additional dwellings per annum (absolute; lower left in Figure S1). The replacement rate of dwellings which were at the end of their service lifetime is tied to reported demolition rates (lower right). These dynamics of the residential housing stock are the basis for the calculation of the material stocks and flows of the residential dwelling stock.

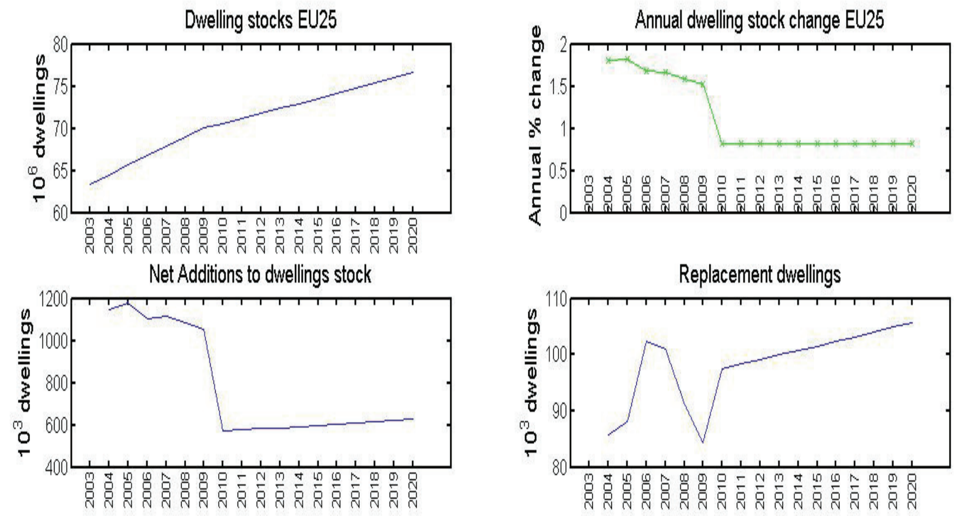

Figure S1: The stocks of dwellings in the EU25 over time (sources see text; own calculations)

Figure S2 and S3 include the country level data on net rates of change dwelling stock change and demolition rates which were used in the model. Details of data compilation are described below and in tables S4-S7.

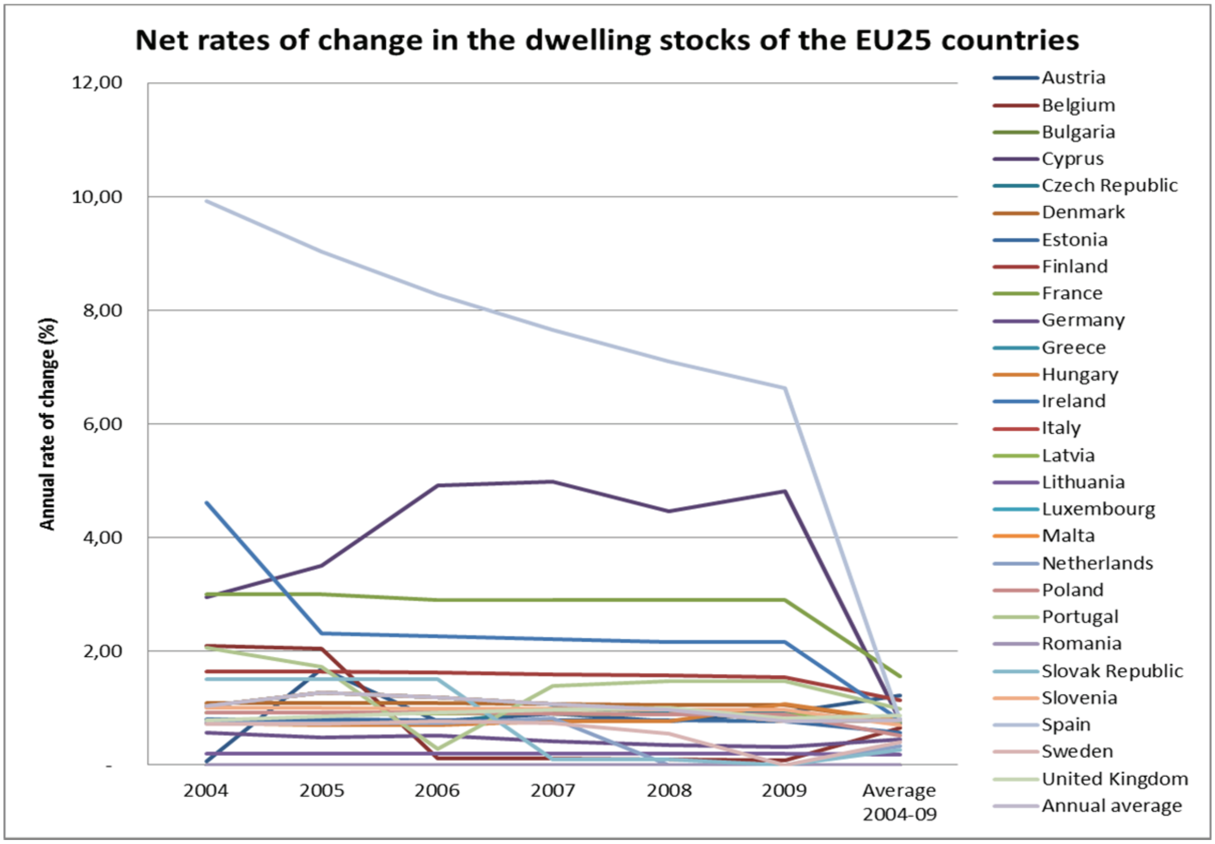

Figure S2: Net rates of change of the EU25 housing stock (data see tables S4-S7)

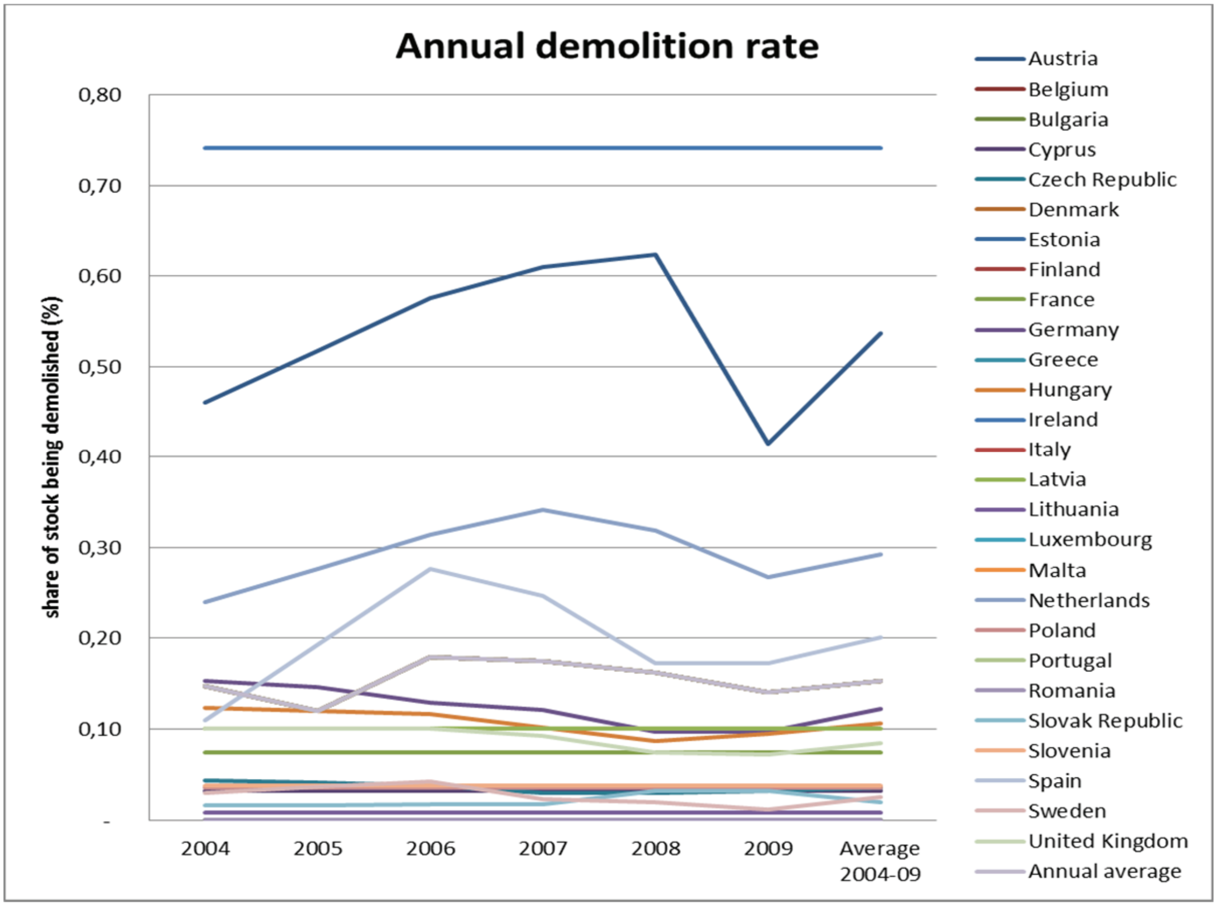

Figure S3: Annual demolition rates of the EU25 housing stock (data see below Tables S4-S7)

The 72 residential housing types

For this study we build on work on residential housing types developed and documented in detail by (Nemry et al., 2008, p. iii). These housing types are the outcome of a European research project, where: “This report [by Nemry et al. 2008] on “Environmental improvement potential of residential buildings” is a scientific contribution of the JRC to the European Commission’s Integrated Product Policy framework which seeks to minimise the environmental degradation caused the life cycle of products. [...] This report presents a systematic overview of the environmental life cycle impacts of residential buildings in EU-25. It also provides an analysis of the technical improvement options that could be help reducing these environmental impacts, with a special focus to their main source, namely energy use for space heating. The report assesses the environmental benefits and the costs associated with these improvement options.”. A summary of the results of this project was also published in the peer-reviewed literature (Nemry et al., 2010). Building on European and

country-level statistics, technical literature and expert interviews, each country's building stock was assessed by the respective time periods they were built in (Figure S4).

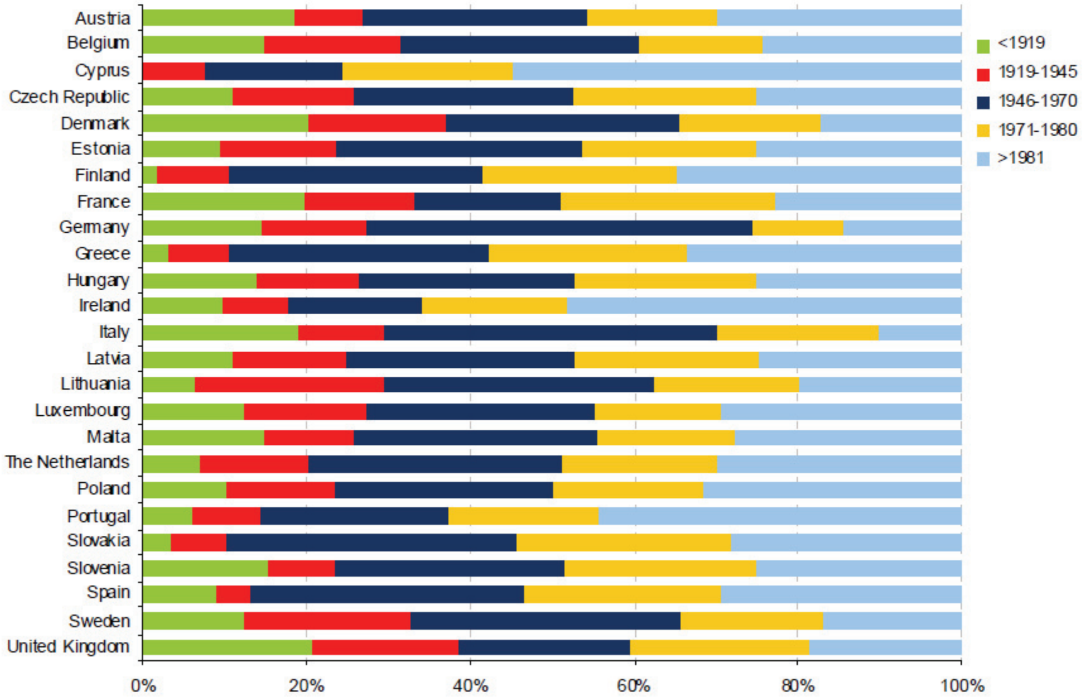

Figure S4: Age structure of the EU25 residential housing stock, in the year 2003, data compiled by (Nemry et al., 2008, p. 11)

Then 'representative' typical buildings were developed for each age class, countries and single vs multi-family and high-rise building (Table S2). These building types include 31 types of single-family buildings, 32 multi-family buildings and 9 different high rise building types, specific for northern, central and southern Europe (classified by heating degree days (Nemry et al., 2008, p. 15)).

Table S2: Number of building types for each climatic zone (reproduced from Nemry et al., 2008, p. 16)

Climatic zones	Single-family house	Multi-family house	High-rise building
Zone 1: South European countries	11 (3)[a]	11 (3)	3 (1)
Zone 2: Central European countries	11 (3)	11 (3)	3 (1)
Zone 3: North European countries	9 (2)	10 (2)	3 (1)

a) Numbers in bracket indicate new building types

Each of the 72 housing types defined by Nemry et al. (2008) are represented in the following manner (Figure S5). Z2 stands for the central European zone, SI represents single family house, 'ex' stands for existing type, whereas the Z2_SI_006 would be version built after 2006, including improved insulation standards. This datasheet contains information on the structural and non-structural composition of the housing type, as well as statistical data on the number of buildings in each country.

Annex C 31   Building type Z2_SI_006_ex

Single-family house
Brick wall, reinforced concrete flooring, pitched roof

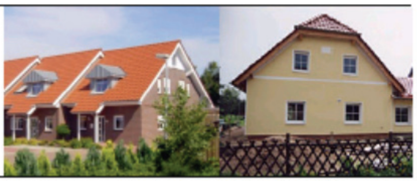

Statistics Proportion of Z2_SI_006_ex in the EU-25: 4.3%

	Belgium	Germany	Luxembourg	The Netherlands	Denmark	Ireland	United Kingdom	Austria	Poland	Slovakia	Slovenia	Czech Republic	Hungary
Number of dwellings [1 000]	816.0	3890.0	20.0	1020.0	130.0	240.0	1280.0	330.0	590.0	95.0	40.0	132.0	205.0
Number of buildings [1 000]	544.0	2593.3	13.3	680.0	86.7	160.0	853.3	220.0	393.3	63.3	26.7	88.0	136.7
Stock in Mio. m^2	70	349	3	100	14	25	111	31	40	5	3	10	15
Density in m^2/occupant	36.0	40.8	50.0	40.8	49.6	36.0	38.0	39.1	22.7	21.6	30.0	30.5	30.0
Occupants per building	3.6	3.3	3.8	3.6	3.3	4.0	3.0	3.6	4.5	3.9	3.8	3.8	4.0

Description of the building type	EXISTING
Zone	2
Building type	Single-family house
Number	006_ex
Year of construction	Since 1980
Residual service life	40 a
Dimension	10 m * 9 m
Storey	1 to 2
Floor to floor height	3 m
Roof	Pitched roof 45°
Roof cladding	Brick
Exterior wall	Brick masonry 25 cm (10 cm insulation)
Interior load-bearing wall	Brick masonry 20 cm
Interior wall	Plasterboard 10 cm
Plaster	Exterior plaster: lime-cement; interior plaster: lime-gypsum
Floor	Reinforced concrete
Basement wall	Reinforced concrete
Basement ceiling	Reinforced concrete
Foundation	Concrete
Window	Plastic frame and double-glazing

Figure S5: Example of a residential building type from (Nemry et al., 2008, p. A236)

Because (Nemry et al., 2008, p. 43) were interested in thermal improvement potentials of each building type, even more detailed structural information was required on the specific materials and for which purposes they were used (eg. If there are bricks or concrete for walls, how thick they are, if stones or tiles are used for floors, if there are plastered ceilings, which materials are structural (load-bearing) and which are non-structural (plastering, tiles, ..). Via a building simulation model, the specific U-values of each of the buildings components were then estimated and the improvement potentials assessed. Figure S6 shows such a detailed description for one specific building type as found in (Nemry et al. 2008). For each of the 72 building types such a data table is available (annex 1 in the report).

Zone	Type and number	Construction/ description	Material	Residual Service Life	Refurbishment Factor	Density (kg/m³)	Thickness (m)	Area (m²)	Volume (m³)	Piece	Mass (kg, St)	Mass (t)
**Z2**	**SI_006_ex**	**Brick masory insulated, reinforced concrete flooring, pitched roof**										
Building's service life: 40		Exterior wall	exterior plaster (lime-cement)	20	1.0	1300	0.02	220	4.4		5720	5.7
			insulation	30	0.3	80	0.1	220	22		1760	1.8
			cored brick	80	0.0	1200	0.35	220	77		92400	92.4
			interior plaster (lime-gypsum)	30	0.3	1000	0.02	220	4.4		4400	4.4
		Interior load-bearing wall	interior plaster (lime-gypsum)	30	0.3	1000	0.02	60	1.2		1200	1.2
			cored brick	80	0.0	1200	0.3	60	18		21600	21.6
			interior plaster (lime-gypsum)	30	0.3	1000	0.02	60	1.2		1200	1.2
		Interior wall	plaster board (gypsum)	20	1.0	1400	0.01	100	1.2		1680	1.7
			wooden construction	20	1.0	500	0.08	10	0.8		400	0.4
			plaster board (gypsum)	20	1.0	1400	0.01	100	1.2		1680	1.7
		Roof	wooden joist (timber spruce 12%), distance 0,6mx0,1	40	0.0	500	0.16	22	3.5		1750	1.8
			mineral insulation	30	0.3	80	0.16	120	19.2		1536	1.5
			roof battening (timber spruce 12%)	25	0.6	500	0.04	13	0.5		250	0.3
			roof tile	25	0.6	2000	0.02	120	2.4		4800	4.8
			NO additional insulation								0	
		Floor	anhydrite screed	30	0.3	2000	0.03	90	2.25	2	9000	9.0
			insulation	30	0.3	80	0.03	90	2.7	2	432	0.4
			reinforced concrete	40	0.0	2400	0.16	90	14.4	2	69120	69.1
			interior plaster (lime-gypsum)	30	0.3	1000	0.02	90	1.8	2	3600	3.6
		Basement wall	reinforced concrete	40	0.0	2400	0.2	80	16		38400	38.4
		Basement ceiling	anhydrite screed	30	0.3	2000	0.03	90	2.25		4500	4.5
			insulation	30	0.3	80	0.03	90	2.7		216	0.2
			reinforced concrete	40	0.0	2400	0.16	90	14.4		34560	34.6
		Basement ground Floor	concrete	40	0.0	2400	0.1	90	9		21600	21.6
		Foundation	concrete	40	0.0	2400	0.5	25	12.5		30000	30.0
		Window	plastic frame 1mx1,5m (with double-glazing)	10	1.2					22		**351.8**
			REFURBISHMENT: window	25						26.4		

Figure S6: detailed information on building type and construction (Nemry et al., 2008, p. A125)

These detailed tables where then aggregated, based on the information on the building component type, lifetime and tons. Specifically, firstly on the materials level we distinguished between concrete and all other non-metallic minerals (bricks, stones, tiles, plaster, ..). Secondly on the construction type level we distinguished between structural and non-structural use, which means that all materials contained in walls and ceiling and having a “residual” lifetime of 80 years, are counted as being structural. All other materials (which have lifetimes ranging from 10 -30 years) are aggregated into non-structural uses. For example concrete contained in the basement and “interior load-bearing wall” (Figure S3), is counted as structural, while the “interior wall” counts as non-structural. Table S3 shows an example of this aggregation procedure.

The final material intensities and lifetimes compiled from Nemry et al. (2008), can be found in Table S4, Table S5 and Table S6. The different identifiers for the residential building types refer to different age categories and building standards. The subscript _ex denotes current buidling technology, whereas the same identifier without _ex refers to up-to-date building standards from 2006 onwards.

Table S3: Aggregation of detailed housing types from Nemry et al. (2008) to the materials modelled. “Structural use” covers materials in walls and basements (80 years residual lifetimes); non-structural uses includes all interior/exterior uses with residual lifetimes from 10-40 years.

Climate Zone: Z2		House Type: Single Family House (SI)			Age Class: since 1980	
**Building component**	**Materials (detailed)**	**Residual service lifetimes**	**Tons**	**structural use**		**non-structural uses**
				Concrete	Non-metallic minerals	Non-metallic minerals
Exterior wall	exterior plaster (lime-cement)	20	5,7			5,7
	Insulation	30	1,8			1,8
	cored brick	80	92,4		92,4	
	interior plaster (lime-gypsum)	30	4,4			4,4
Interior load-bearing wall	interior plaster (lime-gypsum)	30	1,2			1,2
	cored brick	80	21,6		21,6	
	interior plaster (lime-gypsum)	30	1,2			1,2
Interior wall	plaster board (gypsum)	20	1,7			1,7
	wooden construction	20	0,4			
	plaster board (gypsum)	20	1,7			1,7
	wooden joist	40	1,8			
Roof	mineral insulation	30	1,5			1,5
	roof battening (timber spruce 12%)	25	0,3			
	roof tile	25	4,8			4,8
Floor	anhydrite screed	30	9,0			9,0
	insulation	30	0,4			
	reinforced concrete	40	69,1	69,1		
	interior plaster (lime-gypsum)	30	3,6			3,6
Basement wall	reinforced concrete	40	38,4	38,4		
Basement ceiling	anhydrite screed	30	4,5			4,5
	insulation	30	0,2			
	reinforced concrete	40	34,6	34,6		
Basement ground Floor	Concrete	40	21,6	21,6		
Foundation	Concrete	40	30,0	30,0		
Total building weight [tons]				193,7	114	53,6

Table S4: Material intensities and renovation cycles for Zone 1 housing types (Nemry et al. 2008). Zone 1: Malta, Cyprus, Portugal, Greece, Spain, Italy, France

Zone 1	Single Family Houses											Multi-Family Houses											High-Rise Buildings		
	ZI_SI_001	Z1_SI_002	Z1_SI_003	Z1_SI_004	Z1_SI_005_ex	Z1_SI_005	Z1_SI_oo6_ex	Z1_SI_006	Z1_SI_007_ex	Z1_SI_007	Z1_SI_008	Z1_MF_001	Z1_MF_002	Z1_MF_003	Z1_MF_004_ex	Z1_MF_004	Z1_MF_005	Z1_MF_006_ex	Z1_MF_006	Z1_MF_007	Z1_MF_008_ex	Z1_MF_008	Z1_HR_001_ex	Z1_HR_001	Z1_HR_002
**Number of buidlings EU25 [1,000]**	3,397.0	1,474.4	556.0	3,380.0	5,263.4	168.9	3,250.0	168.9	2,500.6	168.9	1,025.3	249.5	108.4	592.1	156.6	12.1	218.8	163.1	12.0	54.9	54.9	6.8	114.9	4.8	114.5
**Construction period**	until 1900	until 1900	1900	1945-1990	since 1965	since 2006	since 1965	since 2006	since 1950	since 2006	since 1900	1945	1900-1945	1945-1990	since 1950	since 2006	since 1965	since 1945	since 2006	1950-1980	since 1970	since 2006	since 1975	since 2006	1970
	**Material Intensities [metric tons per building]**																								
**Structurally used**																									
Concrete (load-bearing walls, foundations, …)	-	-	-	136	184	184	216	216	184	184	-	-	-	723	1,272	1,272	2,034	1,258	1,258	3,565	2,521	2,521	3,176	3,176	4,530
Non-metallic minerals (load-bearing walls, foundations, …)	399	425	277	94	94	94	95	95	94	94	200	2,295	2,542	757	-	-	-	600	600	-	348	348	-	840	-
**Non-structural use**																									
Non-metallic minerals (non load-bearing bricks, tiles, plaster, …)	39	54	25	65	38	38	34	34	38	38	54	326	326	549	249	249	154	262	262	260	404	404	1,355	555	424
	**Probability of demolition [shares in total demolitions]**																								
	8%	8%	8%	0%	0%	4%	0%	0%	0%	0%	4%	4%	4%	8%	8%	0%	8%	8%	0%	8%	5%	0%	5%	0%	8%
	**Renovation cycles of materials in non-structural applications [years]**																								
Non-metallic minerals (bricks, tiles, plaster, …)	25	30	27	26	27	27	27	27	27	27	25	26	26	26	27	26	25	26	28	26	26	26	27	27	28

Table S5: Material intensities and renovation cycles for Zone 2 housing types (Nemry et al. 2008): Zone 2: Belgium, Netherlands, Ireland, Hungary, Slovenia, Luxembourg, Germany, United Kingdom, Slovakia, Denmark, Czech Republic, Austria, Poland

**Zone 2**	**Single-Family Houses**											**Multi-Family Houses**											**High-Rise Buildings**		
	Z2_SI_001	Z2_SI_002	Z2_SI_003	Z2_SI_004	Z2_SI_005	Z2_SI_006_ex	Z2_SI_006	Z2_SI_007_ex	Z2_SI_007	Z2_SI_008_ex	Z2_SI_008	Z2_MF_001	Z2_MF_002	Z2_MF_003	Z2_MF_004	Z2_MF_005_ex	Z2_MF_005	Z2_MF_006_ex	Z2_MF_006	Z2_MF_007_ex	Z2_MF_007	Z2_MF_008	Z2_HR_001	Z2_HR_002_ex	Z2_HR_002
**Number of buidlings EU25 [1,000]**	7,568.3	4,855.6	1,828.1	900.1	9,765.8	5,858.6	211.6	2,034.1	211.6	1,638.7	211.6	446.9	111.4	577.4	249.4	356.8	11.2	146.7	11.2	217.7	11.2	103.3	81.0	45.5	2.9
**Construction period**	until 1945	until 1900	until 1900	1945-1970	1945-1980	since 1980	since 2006	since 1970	since 2006	since 1980	since 2006	until 1945	until 1900	1945-1980	1945-1965	since 1980	since 2006	since 1980	since 2006	1950-1965	since 2006	1970-1990	1960-1990	since 1970	since 2006
**Material Intensities [metric tons per building]**																									
**Structurally used**																									
Concrete (load-bearing walls, foundations, …)	-	-	-	153.1	184.2	184.2	184.2	184.2	184.2	119.7	119.7	-	-	985.6	1,622.2	985.6	985.6	849.5	849.5	2,073.4	2,073.4	3,401.6	4,185.3	2,311.8	2,311.8
Non-metallic minerals (load-bearing walls, foundations, …)	384.3	343.4	200.0	100.8	114.0	114.0	114.0	100.8	100.8	-	-	2,547.0	2,220.4	625.2	-	625.2	625.2	705.6	705.6	-	-	-	-	-	1,368.0
**Non-structural use**																									
Non-metallic minerals (non load-bearing bricks, tiles, plaster, …)	54.4	54.4	54.4	52.5	37.8	39.3	39.3	39.3	39.3	32.2	32.2	326.4	326.4	249.4	241.8	249.4	249.4	229.2	229.2	226.6	226.6	404.3	476.3	10.8	567.3
**Probability of demolition [shares in total demolitions]**																									
	8%	8%	8%	6%	4%	4%	0%	4%	0%	6%	0%	4%	4%	6%	6%	4%	0%	4%	0%	8%	0%	6%	6%	0%	4%
**Renovation cycles of materials in non-structural applications [years]**																									
Non-metallic minerals (bricks, tiles, plaster, …)	26	26	27	28	27	27	27	27	27	24	24	26	26	27	27	27	27	27	27	27	27	26	26	26	26

Table S6: Material intensities and renovation cycles for Zone 3 residential buildings (Nemry et al. 2008). Zone 3: Lithuania, Latvia, Estonia, Sweden, Finland

Zone 3	Single-Family Houses									Multi-Family Houses										High-Rise Buildings		
	Z3_SI_001	Z3_SI_002	Z3_SI_003	Z3_SI_004	Z3_SI_005	Z3_SI_006_ex	Z3_SI_006	Z3_SI_007_ex	Z3_SI_007	Z3_MF_001	Z3_MF_002	Z3_MF_003	Z3_MF_004	Z3_MF_005	Z3_MF_006_ex	Z3_MF_006	Z3_MF_007_ex	Z3_MF_007	Z3_MF_008	Z3_HR_001	Z3_HR_002_ex	Z3_HR_002
**Number of buidlings EU25 [1,000]**	228.7	462.0	209.4	272.0	576.0	232.7	21.8	388.1	21.8	43.1	60.9	32.1	52.2	59.7	13.7	2.7	23.1	1.5	7.6	1.2	1.8	0.1
**Construction period**	until 1945	1945-1980	until 1945	1940-1970	1945-1970	since 1980	since 2006	since 1960	since 2006	until 1940	1940-1980	1940-1970	1945-1980	1960-1990	since 1970	since 2006	since 1980	since 2006	1960-1990	1950-1990	since 1960	since 2006
**Material Intensities [metric tons per building]**																						
**Structurally used**																						
Concrete (load-bearing walls, foundations, …)	-	211.0	-	211.0	195.4	211.0	211.0	119.7	119.7	-	1,633.0	1,100.3	1,478.2	2,000.3	199.2	199.2	985.6	985.6	3,401.6	4,185.3	2,311.8	2,311.8
Non-metallic minerals (load-bearing walls, foundations, …)	384.3	105.6	153.9	26.4	-	66.0	66.0	-	-	2,547.0	-	-	412.8	-	-	-	522.0	522.0	-	-	1,368.0	1,368.0
**Non-structural use**																						
Non-metallic minerals (non load-bearing bricks, tiles, plaster, …)	54.4	37.8	41.9	37.8	46.1	39.3	39.3	32.2	32.2	326.4	244.6	318.1	249.4	221.0	150.3	150.3	229.4	249.4	404.3	476.3	567.3	567.3
**Probability of demolition [shares in total demolitions]**																						
	5%	6%	6%	9%	9%	5%	0%	5%	0%	5%	9%	6%	6%	0%	5%	5%	5%	0%	6%	5%	5%	0%
**Renovation cycles of materials in non-structural applications [years]**																						
Non-metallic minerals (bricks, tiles, plaster, …)	26	27	28	27	26	29	27	24	24	26	27	26	27	27	25	25	27	27	26	26	26	26

Estimating growth and demolition rates for the EU25 housing stocks

To arrive at parameters on growth and demolition for the national housing stocks, several steps were required to overcome the generally quite incomplete datasets. Firstly, from the dwelling types presented above (Nemry et al. 2008), estimates of the total numbers of each housing type in each country for the year 2003 are available (Table S4; Table S5; Table S6). Secondly, a time series of the total number of dwellings in the residential housing stocks of each EU25 member country was compiled from the ‘Housing Statistics in the European Union 2010’ report (Statistics Netherlands, 2012), complemented by national level sources where applicable (Central Statistics Office Ireland, 2012; Statistical Office of the Republic of Slovenia, 2012; Statistical Service Cyprus, 2012; Statistics Austria, 2012; Statistics Netherlands, 2012; UK National Statistics, 2012). The compiled data is presented for 2003 – 2009 in (Table S7), with some large gaps remaining. All freely available statistical national online databases of the remaining EU25 countries were consulted, with limited avail (Table S7; see colouring of cells). Secondly, also from the above mentioned sources, the category “dwellings completed by 1,000 inhabitants” as well as “Dwellings demolished or otherwise removed from the housing stock” was compiled (Figure S7; Table S9; Table S10;).Using population numbers from Eurostat, the absolute numbers for completed and demolished dwellings were calculated and used to calculate the dwelling stocks for the remaining years (Table S7 cells in purple; last existing stock data + dwellings completed – dwellings demolished). Thirdly, for a number of countries not enough data was available to follow this procedure, due to one of the three components (stock, demolished and finished dwellings) missing. Then a second-best solution was applied, where the gaps in the stock data were linearly interpolated (Table S7; cells in yellow). Even then not for all countries a full time series from 2003 – 2009 could be compiled. From the compiled dwelling stock dataset (Table S7) annual rates of change were calculated (Table S9), where yellow cells indicate extrapolation from the last existing data point and green cells indicate that the EU25 average (of the other existing datapoints) was used, to overcome the lack of data. Finally, based on the compiled dwelling stock dataset (Table S7) and the absolute numbers of demolished dwellings, annual demolition rates were estimated (Table S10). For those cases where no annual rates were computable, the last existing data point of the country was used (yellow cells). For those countries where no data was available at all, the annual EU25 average was used (green cells; Table S10). Overall the demolition rates agree well with data presented in the literature (Deilmann et al., 2009; Thomsen and van der Flier, 2009). (Deilmann et al., 2009)For the trend scenario until 2020 the averages of the existing data on demolition and finished dwellings were used (right-most column of Table S9; Table S10). Because for this business-as-usual trend scenario at least the effects of the financial crisis have to be taken into account,

all average growth rates of the dwelling stock which were larger than 2% (Cyprus, France, Ireland, Spain), were reduced to the average of the remaining EU25 countries, which is 0.78 % (average calculated without the cases >2%).

Table S7: National dwelling stocks for the EU25 countries (sources see text; purple cells are estimated from the last existing data point + new dwellings – demolished dwellings; yellow cells are linearily interpolated; other colours refer to the national level statistical sources, see text. This dataset is only used for the estimation of national level demolition & growth rates

[1,000 dwellings]	2003	2004	2005	2006	2007	2008	2009
Austria	3,429.0	3,431.4	3,489.7	3,516.3	3,548.4	3,575.9	3,608.6
Belgium	4,820.0	4,920.9	5,021.8	5,027.9	5,033.3	5,038.5	5,043.0
Cyprus	305.0	314.0	325.0	341.0	358.0	374.0	392.0
Czech Republic	3,853.7	3,884.0	3,912.0	3,940.2	3,980.2	4,017.4	4,054.8
Denmark		2,541.0	2,568.8	2,596.6	2,624.4	2,652.2	2,680.0
Estonia		626.0	631.0	636.0	641.0	646.0	651.0
Finland		2,572.0	2,614.4	2,656.8	2,699.2	2,741.6	2,784.0
France	28,858.0	29,495.0	30,379.5	31,264.0	31,430.2	31,596.3	31,264.0
Germany	39,141.5	39,362.3	39,551.2	39,753.7	39,918.2	40,057.3	40,183.6
Greece							
Hungary	4,103.7	4,134.0	4,163.3	4,192.6	4,224.8	4,257.2	4,303.0
Ireland	1,547.5	1,619.0	1,656.5	1,694.0	1,731.5	1,769.0	
Italy		26,526.0					
Latvia	987.0	996.2	1,005.3	1,014.5	1,023.7	1,032.8	1,042.0
Lithuania	1,292.0	1,294.7	1,297.3	1,300.0	1,302.7	1,305.3	1,308.0
Luxembourg							188.0
Malta			139.0				-
Netherlands		6,810.0	6,862.9	6,913.0	6,969.4	7,025.7	7,107.0
Poland		12,683.0	12,799.8	12,916.5	13,033.3	13,150.0	
Portugal	5,318.0	5,427.5	5,520.9	5,537.0	5,614.4	5,697.2	-
Slovak Republic		8,176.0	8,189.2	8,313.0	8,321.2	8,329.0	-
Slovenia		798.0	806.0	814.0	822.0	830.0	838.0
Spain	15,747.6	17,311.1	18,874.7	20,438.3	22,001.9	23,565.4	25,129.0
Sweden		4,380.0	4,411.6	4,445.2	4,478.3	4,503.0	4,503.0
United Kingdom	21,513.0	21,684.0	21,870.0	22,073.0	22,288.0	22,511.0	22,694.0

Growth of the dwelling stock

The procedures to match the rates of annual net stock change (Table S9), with the dwelling types data was the following: Firstly, annual rates of stock change per country $r_{c,y}$ (Table S9) were used to estimate the absolute change (increase) of each dwelling stock type $DwellsChange_{c,y,DT}$.

$$DwellsChange_{c,y,DT} = dwells_{c,y,DT} * r_{c,y}$$

Stock degrowth was deducted from the total stock $dwells_{c,y,DT}$, (which based on the data was not the case, see Table S7 and Table S9) while all dwelling stock increase was shifted to those dwelling types which are current building standards, i.e. to those for single family, multi-family and high-rise buildings (Table S2). The underlying assumption would be, that a single-family house from the 1960's, which was demolished, will be replaced by a single-family house of today's standards.

Table S8: Dwellings completed (left part of the table; 1,000) and dwellings demolished (right part of the table; 1,000), as compiled from (sources see text); blue cells are the same as their neighbouring cell; green cells are averages of the neigbhouring cells

	2004	2005	2006	2007	2008	2009	2004	2005	2006	2007	2008	2009
Austria	42.0	-	46.8	53.7	49.8	47.7	15.8	-	20.2	21.6	22.3	15.0
Belgium	46.0	-	64.1	57.2	55.5	48.4	-	-	-	-	-	-
Cyprus	11.01	16.4	16.6	16.5	18.2	16.6	0.1	-	-	-	-	-
Czech Republic	32.0	29.6	29.7	41.1	38.4	38.7	1.7	1.6	1.5	1.2	1.2	1.3
Denmark	25.0	-	28.8	31.0	25.7	16.0	-	-	-	-	-	-
Estonia	2.0	-	5.1	7.1	5.4	3.1	-	-	-	-	-	-
Finland	31.0	-	33.6	35.4	30.2	21.8	-	-	-	-	-	-
France	363.0	-	448.9	477.3	473.7	462.0	22.0	-	-	-	-	-
Germany	239.6	214.0	219.8	159.7	150.2	154.8	60	58	51	48	39	36
Greece	122.0	195.2	125.4	-	-	62.0	6.3	-	7.1	6.1	5.0	4.1
Hungary	44.0	34.3	34.3	36.2	36.2	32.1	5.1	5.0	4.9	4.1	3.7	4.1
Ireland	77	81.0	93.4	78.0	51.7	-	12.0	-	-	-	-	-
Italy	256.0	-	-	-	-	-	-	-	-	-	-	-
Latvia	1.0	-	6.0	9.4	8.2	8.0	1.0	-	-	-	-	-
Lithuania	5.0	-	-	-	-	-	0.1	-	-	-	-	-
Luxembourg	2.0	1.0	2.3	3.0	1.7	1.5	-	-	-	-	-	-
Malta	-	-	-	-	-	-	0.1	-	-	-	-	-
Netherlands	-	71.9	71.9	80.2	78.7	82.4	16.3	19.0	21.7	23.8	22.4	19.0
Poland	108.0	-	114.5	133.4	163.9	160.2	4.5	-	-	-	-	-
Portugal	82.0	-	70.8	77.4	82.8	80.0	-	-	-	-	-	-
Slovak Republic	13.0	14.6	14.6	16.7	17.3	18.9	1.3	1.4	1.4	1.4	2.7	1.2
Slovenia	-	11.7	13.7	16.2	13.7	11.1	0.3	-	-	-	-	-
Spain	544.0	-	638.9	622.6	611.3	424.0	19.0	-	56.6	54.2	40.7	-
Sweden	30.0	-	33.5	34.6	34.0	24.1	1.3	-	1.9	1.0	0.9	0.5
United Kingdom	206.0	-	193	200	158	124	-	-	22	21	17	16

Table S9: Annual rates of change for the residential dwelling stock (sources see text; yellow cells are extrapolation from the last existing data point; green cells are the EU25 average due to lacking data)

[%]	2004	2005	2006	2007	2008	2009	Average 2004-09
Austria	0.07	1.70	0.76	0.91	0.77	0.91	1.22
Belgium	2.09	2.05	0.12	0.11	0.10	0.09	0.65
Cyprus	2.95	3.50	4.92	4.99	4.47	4.81	3.95
Czech Republic	0.79	0.72	0.72	1.01	0.93	0.93	0.73
Denmark	1.09	1.09	1.08	1.07	1.06	1.05	0.76
Estonia	0.80	0.80	0.79	0.79	0.78	0.77	0.56
Finland	1.65	1.65	1.62	1.60	1.57	1.55	1.14
France	3.00	3.00	2.91	2.91	2.91	2.91	1.56
Germany	0.56	0.48	0.51	0.41	0.35	0.32	0.46
Greece	1.04	1.27	1.18	1.08	0.97	0.78	0.78
Hungary	0.74	0.71	0.71	0.77	0.77	1.08	0.79
Ireland	4.62	2.32	2.26	2.21	2.17	2.17	2.63
Italy	1.04	1.27	1.18	1.08	0.97	0.78	0.78
Latvia	0.93	0.92	0.91	0.90	0.90	0.89	0.78
Lithuania	0.21	0.21	0.21	0.21	0.20	0.20	0.18
Luxembourg	1.04	1.27	1.18	1.08	0.97	0.78	0.78
Malta	1.04	1.27	1.18	1.08	0.97	0.78	0.78
Netherlands	0.73	0.73	0.73	0.82	-	-	0.33
Poland	0.92	0.92	0.92	0.90	0.90	0.90	0.52
Portugal	2.06	1.72	0.29	1.40	1.48	1.48	0.99
Slovak Republic	1.51	1.51	1.51	0.10	0.09	-	0.27
Slovenia	1.00	1.00	0.99	0.98	0.97	0.96	0.70
Spain	9.93	9.03	8.28	7.65	7.11	6.64	8.52
Sweden	0.72	0.72	0.76	0.74	0.55	-	0.40
United Kingdom	0.79	0.86	0.93	0.97	1.00	0.81	0.87
Annual average	1.04	1.27	1.18	1.08	0.97	0.78	1.12

Table S10: Demolition rates for the EU25 dwelling stock (sources see text; yellow cells are extrapolation from the last existing data point; green cells are the EU25 average due to lacking data)

	2004	2005	2006	2007	2008	2009	Average 2004-09
Austria	0.46	0.52	0.58	0.61	0.62	0.41	0.54
Belgium	0.15	0.12	0.18	0.18	0.16	0.14	0.15
Cyprus	0.03	0.03	0.03	0.03	0.03	0.03	0.03
Czech Republic	0.04	0.04	0.04	0.03	0.03	0.03	0.04
Denmark	0.15	0.12	0.18	0.18	0.16	0.14	0.15
Estonia	0.15	0.12	0.18	0.18	0.16	0.14	0.15
Finland	0.15	0.12	0.18	0.18	0.16	0.14	0.15
France	0.07	0.07	0.07	0.07	0.07	0.07	0.07
Germany	0.15	0.15	0.13	0.12	0.10	0.10	0.12
Greece	0.15	0.12	0.18	0.18	0.16	0.14	0.15
Hungary	0.12	0.12	0.12	0.10	0.09	0.10	0.11
Ireland	0.74	0.74	0.74	0.74	0.74	0.74	0.74
Italy	0.15	0.12	0.18	0.18	0.16	0.14	0.15
Latvia	0.10	0.10	0.10	0.10	0.10	0.10	0.10
Lithuania	0.01	0.01	0.01	0.01	0.01	0.01	0.01
Luxembourg	0.15	0.12	0.18	0.18	0.16	0.14	0.15
Malta	0.15	0.12	0.18	0.18	0.16	0.14	0.15
Netherlands	0.24	0.28	0.31	0.34	0.32	0.27	0.29
Poland	0.04	0.04	0.04	0.04	0.04	0.04	0.04
Portugal	0.15	0.12	0.18	0.18	0.16	0.14	0.15
Slovak Republic	0.02	0.02	0.02	0.02	0.03	0.03	0.02
Slovenia	0.04	0.04	0.04	0.04	0.04	0.04	0.04
Spain	0.11	0.19	0.28	0.25	0.17	0.17	0.20
Sweden	0.03	0.04	0.04	0.02	0.02	0.01	0.03
United Kingdom	0.10	0.10	0.10	0.09	0.07	0.07	0.08
Annual average	0.15	0.12	0.18	0.18	0.16	0.14	0.15

Road and rail network: Data sources and compilation procedures

For the road and rail network a combination of various sources was used to compile a complete dataset of infrastructure extent (in km) by type (4 road and 2 railway types) for each country for the time period of interest (see appendix).

Infrastructure extent and temporal dynamics

The primary source for both railway and road extent data is (Eurostat, 2011). Gaps in the time series were filled with data from the European Road Federation and the United Nations Economic Commission for Europe (ERF, 2007 in green; ERF et al., 2010 in grey; UNECE, 2011 in yellow). In Germany municipal roads, which amount to approximately 430,000 km, or 2/3 of the total network, were last surveyed in 1993 and are usually not included in the official Eurostat statistics (Reichel, 2012). For the purpose of this study these 430,000km were added to communal roads as a constant. The country-level time series were used to estimate the trend for infrastructure extent change. The average of the annual rate of change over the whole time period for each country and infrastructure type was taken. Remaining gaps in the time series were either interpolated were only years in between were missing, or extrapolated using this averaged rate of change (all cells in orange in tables S1 – S6). This averaged rates of change (tables S1-S6 right hand side) were also used for the ceteris paribus trend scenario until the year 2020. The resulting time series of motorways, state roads, provincial roads and communal roads as well as single track and double or more track railway lines for each EU25 country, from 1995-2009 are shown in (tables S1-S6).

In 2009 the majority of the road and railway network of the EU25 was composed of communal roads, followed by provincial roads (Figure S7). The railway network (216 thousand km) is extremely small compared to the roads network (5,264 thousand kilometer). It has to be noted that this infrastructure datasets does not cover bridges, tunnels, intra-city railways or subways.

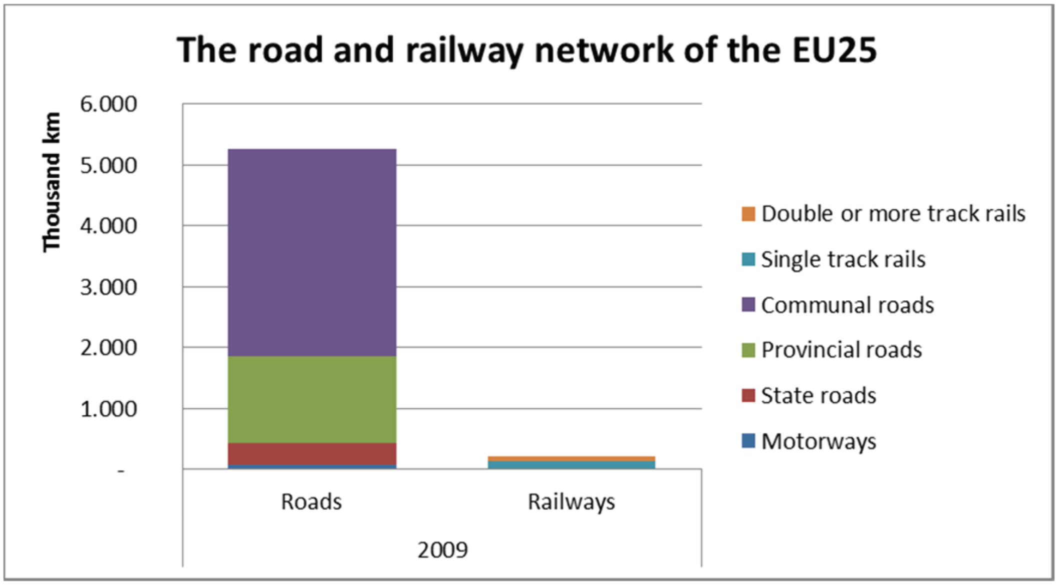

Figure S7: The composition of the EU25 road and railway network in 2009 (data from tables S1-S6; sources see text)

Over the whole time period the road and railway network in the EU25 is steadily being expanded (Figure S8). The extent of motorways, provincial and communal roads, as well as double or more track railway lines is growing steadily. Only for state roads and single track railway lines slight decreases of extent can be observed. At least to some extent these changes are also due to upgrading of roads and railway lines, i.e. expanding a single track railway line into a double track line, or reconstructing a state road into a motorway. Also some of the annual changes are probably due to data and statistical problems, where for in some countries roads are reclassified from one year to the next.

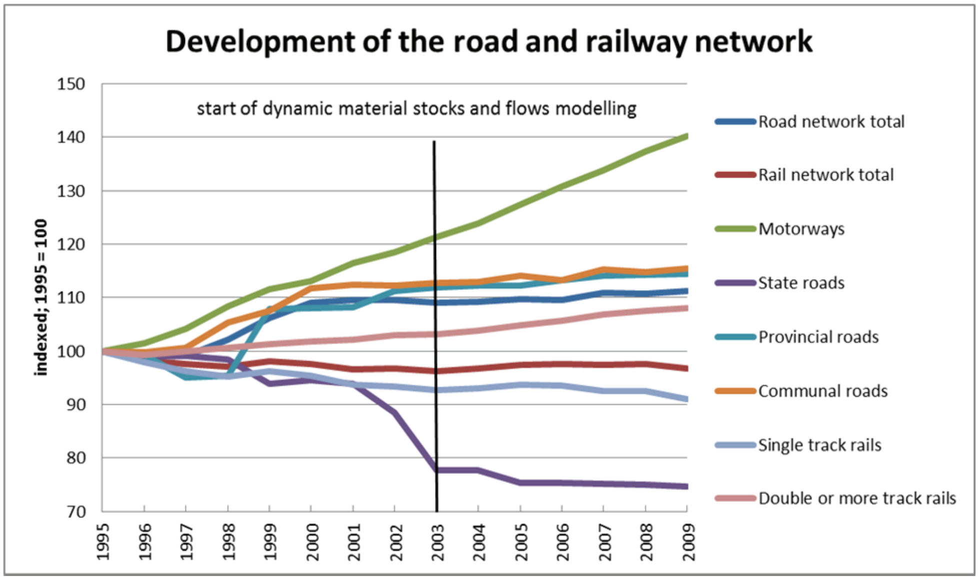

Figure S8: Development of the EU25 road and railway network (indexed data from Table S1 – S6).

Material composition of road and railway infrastructure

As a next step average material intensities for a given kilometer of roads and railways were compiled from various literature sources, for each infrastructure type (Table S11). Roads in this context are separated into a subsurface foundation layer, mostly consisting of gravel, sand, crushed rocks and recycled construction & demolition waste. The carrying layer for roads is made from asphalt and concrete (the later mostly in motorways). The average over these sources for each infrastructure type was used in the modelling exercise (Table S11).

Table S11: The material intensities of infrastructure types in the EU25

		Asphalt, concrete	Gravel,sand,fillings
**Motorways**	Average (tons / km)	14,341	25,864
	(Eurostat, 2012)		28,383
	(Tanikawa and Hashimoto, 2009)	19,130	24,010
	(Birgisdottir et al., 2006)		
	(Rubli et al., 2005)	9,594	
	(Unseld, 2003)	14,300	25,200
**State roads**	Average (tons / km)	3,489	8,011
	(Eurostat, 2012)		9,692
	(Tanikawa and Hashimoto, 2009)	4,066	5,773
	(Birgisdottir et al., 2006)		
	(Rubli et al., 2005)	2,516	
	(Unseld, 2003)	3,887	8,568
**Provincial roads**	Average (tons / km)	2,932	6,280
	(Eurostat, 2012)		8,719
	(Tanikawa and Hashimoto, 2009)	2,432	3,822
	(Rubli et al., 2005)		
	(Unseld, 2003)	3,432	6,300
**Communal roads**	Average (tons / km)	1,393	4,208
	(Eurostat, 2012)		6,253
	(Tanikawa and Hashimoto, 2009)	798	1,872
	(Rubli et al., 2005)	1,509	
	(Unseld, 2003)	1,872	4,500
**Railways (intensities refer to 1 track; 2 track = doubled)**	Average (tons / km)	308	3,419
	(Tanikawa and Hashimoto, 2009)		3,000
	(Rubli et al., 2005)	108	8,400
	(Stripple and Uppenberg, 2010)	417	487
	(Milford and Allwood, 2010)	416	1,890
	(Unseld, 2003)	290	3,317

Service lifetimes of the road and railway infrastructure

Regarding the average service lifetimes and maintenance cycles the literature yields mixed indications. Generally those roads, which are heavily used are subject to more frequent maintenance and renewal activities, while lower order roads (like communal roads) are less frequently maintained. Additionally mostly the surface layer, consisting of asphalt, concrete and bitumen is subject to maintenance, while the foundation remains in place. This is also reflected in the lifetimes compiled from the literature (Table S12). For the surface layers of communal, provincial and state roads, based on the literature, 20 years as lifetime have been choosen, while motorways, which are subject to much more intensive use, 17 years are used. The foundational structures have lifetimes of 60 years. For the railway system a rather high value of 47,5 years has been compiled from the literature, which relates to the concrete sleepers which are widely used, whereas the basements and groundations of the railway track are treated with 60 years lifetimes. As mentioned above, neither of the transportation infrastructure estimates include additional constructions like bridges, tunnels as well as special obstructions required in mountainous areas.

Table S12: Service lifetimes of road and railway infrastructure, by material, compilation of literature

		Asphalt, concrete	Gravel,sand,fillings
**Motorways**	Average lifetime (years for km and material)	17	60
	(Christensen, 2012)	10	
	(Huang et al., 2009)	15	
	(Hashimoto et al., 2007)	20	
	(Birgisdottir et al., 2006)	20	
	(Cochran and Townsend, 2010)	20	
			60
**State roads**	Average lifetime (years for km and material)	20	60
	(Hashimoto et al., 2007)	20	
	(Birgisdottir et al., 2006)	20	
	(Cochran and Townsend, 2010)	20	
			60
**Provincial roads**	Average lifetime (years for km and material)	20	60
	(Hashimoto et al., 2007)	20	
	(Birgisdottir et al., 2006)	20	
	(Cochran and Townsend, 2010)	20	
			60
**Communal roads**	Average lifetime (years for km and material)	20	60
	(Birgisdottir et al., 2006)	20	
	(Cochran and Townsend, 2010)	20	
			60
**Railways**	Average lifetime (years for km and material)	47.3	60
	(Stripple and Uppenberg, 2010)		60

(Manalo et al., 2010)	60
(Milford and Allwood, 2010)	34.5

Literature

Birgisdottir, H., Pihl, K.A., Bhander, G., Hauschild, M.Z., Christensen, T.H., 2006. Environmental assessment of roads constructed with and without bottom ash from municipal solid waste incineration. Transp. Res. Part -Transp. Environ. 11, 358–368.

Central Statistics Office Ireland, 2012. Construction And Housing In Ireland 2008 Edition [WWW Document]. URL http://www.cso.ie/en/newsandevents/pressreleases/2008pressreleases/constructionandhousinginireland2008edition/ (accessed 8.6.12).

Christensen, J., 2012. Economic Evaluation of Long-Life Pavements (Phase 1).

Cochran, K.M., Townsend, T.G., 2010. Estimating construction and demolition debris generation using a materials flow analysis approach. Waste Manag. 30, 2247–2254.

Deilmann, C., Effenberger, K.-H., Banse, J., 2009. Housing stock shrinkage: vacancy and demolition trends in Germany. Build. Res. Inf. 37, 660–668.

ERF, 2007. European Road Statistics 2007. European Commission, IRF, UN, National Road Associations, Brussels, Belgium.

ERF, Nicodeme, C., Diamandouros, K., Diez, J.L., Fusco, I., Minarro, M.L.L., 2010. European Road Statistics 2010. European Union Road Federation, Brussels, Belgium.

Eurostat, 2011. http://epp.eurostat.ec.europa.eu/portal/page/portal/transport/data/main_tables.

Eurostat, 2012. Economy-wide Material Flow Accounts (EW-MFA). Compilation Guide 2012. Eurostat.

Hashimoto, S., Tanikawa, H., Moriguchi, Y., 2007. Where will large amounts of materials accumulated within the economy go? - A material flow analysis of construction minerals for Japan. Waste Manag. 27, 1725–1738.

Huang, Y., Bird, R., Heidrich, O., 2009. Development of a life cycle assessment tool for construction and maintenance of asphalt pavements. J. Clean. Prod. 17, 283–296.

Manalo, A., Aravinthan, T., Karunasena, W., Ticoalu, A., 2010. A review of alternative materials for replacing existing timber sleepers. Compos. Struct. 92, 603–611.

Milford, R.L., Allwood, J.M., 2010. Assessing the CO2 impact of current and future rail track in the UK. Transp. Res. Part -Transp. Environ. 15, 61–72.

Nemry, F., Uihlein, A., Colodel, C.M., Wetzel, C., Braune, A., Wittstock, B., Hasan, I., Kreißig, J., Gallon, N., Niemeier, S., Frech, Y., 2010. Options to reduce the environmental impacts of residential buildings in the European Union—Potential and costs. Energy Build. 42, 976–984.

Nemry, F., Uihlein, A., Makishi Colodel, C., Wittstock, A.B., Wetzel, C., Hasan, I., Niemeier, S., Frech, Y., Kreissig, J., Gallon, N., 2008. Environmental Improvement Potential of Residential Buildings. Joint Research Center.

Reichel, B., 2012. personal communication via e-mail: German Statistical Agency, Customer number 296560 / 391585.

Rubli, S., Werkstoff-Börse GmbH, Jungbluth, N., ESU-services, 2005. Materialflussrechnung für die Schweiz. Machbarkeitsstudie. Bundesamt für Statistik (BFS), Neuchâtel.

Statistical Office of the Republic of Slovenia, 2012. Statistical Office of the Republic of Slovenia [WWW Document]. URL http://www.stat.si/eng/novica_prikazi.aspx?id=3456 (accessed 7.5.12).

Statistical Service Cyprus, 2012. Statistical Service - Industry, Construction - Construction - Announcements - New Publication: Construction and Housing Statistics, 2009 [WWW

Document]. URL http://www.mof.gov.cy/mof/cystat/statistics.nsf/All/8593A7CD7C9CD436C2257873002C7F89?OpenDocument&sub=2&sel=1&e=&print (accessed 8.6.12).

Statistics Austria, 2012. Statistics Austria [WWW Document]. URL http://www.statistik.at/web_en/statistics/dwellings_buildings/index.html (accessed 7.6.12).

Statistics Netherlands, 2012. Housing Statistics in the European Union 2010 | Jaargang 2010, Nummer 5, 17 december 2010 | Kennisplein WBI | Archief nieuwsbrieven - Rijksoverheid.nl [WWW Document]. URL http://abonneren.rijksoverheid.nl/article/kennisplein-wwi/nieuwsbrief-kennisplein-wwi-december-2010/housing-statistics-in-the-european-union-2010/428/3384 (accessed 8.6.12).

Stripple, H., Uppenberg, S., 2010. Life cycle assessment of railways and rail transports - application in environmental product declarations (EPDs) for the Bothnia Line (No. IVL Report B1943). Swedish Environmental Research Institute (IVL), Stockholm, Sweden.

Tanikawa, H., Hashimoto, S., 2009. Urban stock over time: spatial material stock analysis using 4d-GIS. Build. Res. Inf. 37, 483–502.

Thomsen, A., van der Flier, K., 2009. Replacement or renovation of dwellings: the relevance of a more sustainable approach. Build. Res. Inf. 37, 649–659.

UK National Statistics, 2012. UK National Statistics [WWW Document]. URL www.statistics.gov.uk (accessed 7.5.12).

UNECE, 2011. United Nations Economic Commission for Europe. Statistical Database [WWW Document]. URL http://w3.unece.org/pxweb/database/STAT/40-TRTRANS/09-TRINFRA/?lang=1 (accessed 11.20.12).

Unseld, B., 2003. Entwicklung von Rohstoffkoeffizienten für den Tiefbau an Hand von Normen und praktischen Erfahrungen, besonders im Bundesland Oberösterreich, für Straßenbau, Eisenbahnbau, Brückenbau und Netzwerke. Montanuniversität Leoben, Department Bergbau und Tunnelbau.

Appendix A: Time series data of road and rail infrastructure extent for all EU25 members from 1990-2009

Table S13: Motorways in the EU25 member states (Eurostat, 2011 in white; UNECE, 2011 in yellow). Orange cells mark those years which were linearly inter/extrapolated (see method discussion above for details)

[km]	1995	1996	1997	1998	1999	2000	2001	2002	2003	2004	2005	2006	2007	2008	2009	Average rate of change from 1995-2009
Austria	1,596	1,607	1,613	1,613	1,634	1,633	1,645	1,645	1,670	1,677	1,677	1,678	1,696	1,696	1,696	0.44%
Belgium	1,666	1,674	1,679	1,682	1,691	1,702	1,727	1,729	1,729	1,747	1,747	1,763	1,763	1,763	1,763	0.41%
Cyprus	168	168	194	204	216	240	257	268	268	268	276	257	257	257	257	1.56%
Czech Republic	414	423	486	499	499	499	518	518	518	546	564	633	657	691	729	2.65%
Denmark	796	832	855	873	892	953	971	1,010	1,027	1,032	1,032	1,071	1,111	1,128	1,158	2.73%
Estonia	65	65	68	74	87	93	93	98	98	96	99	99	96	104	100	2.17%
Finland	394	431	444	473	512	549	591	603	653	653	693	700	700	739	765	4.90%
France	8,275	8,596	8,864	9,303	9,626	9,766	10,068	10,223	10,379	10,486	10,800	10,848	10,958	11,042	11,163	2.17%
Germany	11,190	11,246	11,309	11,427	11,515	11,712	11,786	12,037	12,044	12,174	12,363	12,531	12,594	12,645	12,813	0.97%
Greece	730	730	730	730	730	730	730	730	730	730	880	903	925	948	957	0.63%
Hungary	335	365	382	448	448	448	448	533	542	569	636	785	858	1,274	1,273	3.28%
Ireland	70	80	94	103	103	103	125	125	176	192	247	360	269	423	663	2.27%
Italy	6,435	6,465	6,469	6,478	6,478	6,478	6,478	6,487	6,487	6,532	6,542	6,554	6,588	6,629	6,780	0.23%
Latvia	-	-	-	-	-	-	-	-	-	-	-	-	-	-	-	0.00%
Lithuania	394	404	410	417	417	417	417	417	417	417	417	309	309	309	309	0.44%
Luxembourg	-	-	-	115	115	114	115	126	147	146	147	147	147	147	152	0.38%
Malta	-	-	-	-	-	-	-	-	-	-	-	-	-	-	-	0.00%
Netherlands	2,208	2,272	2,336	2,225	2,291	2,265	2,499	2,516	2,542	2,585	2,600	2,604	2,582	2,637	2,631	1.31%
Poland	246	258	264	268	317	358	398	405	405	552	552	663	663	765	849	1.50%
Portugal	687	710	797	1,252	1,441	1,482	1,659	1,835	2,190	2,190	2,300	2,545	2,613	2,652	2,692	2.78%
Slovakia	19	21	21	29	29	29	29	30	31	31	32	32	36	38	39	2.4

	8	5	9	2	5	6	6	2	3	6	8	8	5	4	1	3%
Slovenia	293	310	330	369	399	427	435	456	477	483	569	579	579	696	747	4.46%
Spain	6,962	7,295	7,750	8,269	8,893	9,049	9,571	9,739	10,296	10,747	11,432	12,073	13,013	13,515	13,844	5.25%
Sweden	1,262	1,350	1,423	1,439	1,484	1,499	1,507	1,544	1,591	1,684	1,677	1,744	1,806	1,855	1,938	3.03%
United Kingdom	3,269	3,298	3,378	3,554	3,582	3,600	3,610	3,611	3,611	3,657	3,629	3,665	3,669	3,673	3,674	0.85%

Table S14: State roads network in the EU25 (ERF, 2007 in grey; Eurostat, 2011 in white; UNECE, 2011 in yellow). Orange cells mark those years which had to be linearly inter/extrapolated (see method discussion above for details)

[km]	1995	1996	1997	1998	1999	2000	2001	2002	2003	2004	2005	2006	2007	2008	2009	Average rate of change from 1995-2009
Austria	10,243	10,269	10,267	10,276	10,260	10,280	10,213	10,145	10,078	10,010	10,193	10,024	9,996	9,993	9,994	-0.17%
Belgium	12,583	12,600	12,509	12,542	12,542	12,550	12,600	12,610	12,531	12,531	12,531	12,585	12,597	12,613	12,760	0.10%
Cyprus	4,855	4,952	5,058	4,959	5,052	5,098	5,261	5,368	5,440	5,573	5,554	5,589	5,332	5,344	5,359	0.73%
Czech Republic	55,086	55,088	54,908	54,895	54,933	54,909	54,910	54,904	6,121	6,156	6,154	6,174	6,191	6,210	6,198	0.07%
Denmark	3,764	3,751	3,780	758	749	718	689	662	649	641	641	610	2,755	2,711	2,656	-2.04%
Estonia	14,922	15,303	16,369	16,356	16,343	16,340	16,341	16,442	16,452	16,459	16,371	16,380	16,369	16,487	16,370	0.68%
Finland	77,328	77,351	77,352	77,421	77,388	77,444	77,468	77,534	77,544	77,515	77,496	77,489	77,461	77,403	77,396	0.01%
France	28,097	26,881	26,856	26,584	26,298	26,126	26,050	26,154	26,127	26,014	9,760	10,365	9,861	9,765	9,768	-4.98%
Germany	41,700	41,487	41,419	41,386	41,321	41,282	41,228	41,246	41,139	40,969	40,983	40,711	40,416	40,203	39,887	-0.32%
Greece	10,189	10,189	10,189	10,189	10,189	10,189	10,189	10,189	10,189	10,189	10,189	9,892	9,596	9,299	9,090	-2.25%
Hungary	29,653	29,357	29,415	29,464	30,267	30,307	30,322	30,460	30,536	30,638	30,808	31,085	31,182	31,363	30,104	0.12%
Ireland	5,260	5,270	5,350	5,329	5,326	5,326	5,310	5,327	5,255	5,230	5,168	5,298	5,428	5,433	5,443	0.25%
Italy	45,130	46,043	45,819	42,977	43,319	46,556	46,870	20,654	17,250	17,250	21,524	21,524	19,290	19,290	19,382	0.48%
Latvia	20,411	20,332	20,332	20,329	20,318	20,323	20,320	20,279	20,309	20,227	20,182	20,167	20,180	20,176	20,178	-0.08%
Lithuania	20,727	20,717	20,711	20,747	20,854	20,896	20,899	20,918	20,916	20,928	20,911	21,016	21,011	21,011	20,959	0.08%
Luxemb	83	83	83	83	83	83	83	83	83	83	83	83	83	83	83	0.0

ourg	3	0	0	7	7	7	7	7	7	9	7	7	7	7	7	3%
Malta					184	184	184	184	184	184	184	184	184	184	184	0.00%
Netherlands	998	985	986	814	893	894	913	932	951	970	989	938	790	751	749	-0.33%
Poland	45,431	45,417	45,384	45,409	17,803	17,706	18,116	18,166	18,253	18,368	18,287	18,439	18,546	18,520	18,579	0.33%
Portugal	9,055	9,032	8,983	10,156	14,099	13,883	10,351	10,564	11,109	11,655	12,200	12,452	12,710	12,973	13,241	2.07%
Slovakia	3,074	3,073	3,219	3,223	3,220	3,222	3,220	3,224	3,335	3,341	3,341	3,359	3,366	3,434	3,496	0.93%
Slovenia	4,752	4,765	4,796	5,797	5,855	5,845	5,897	5,893	5,864	5,866	5,824	5,842	5,897	5,958	5,954	1.75%
Spain	17,294	17,266	17,169	17,132	17,195	17,123	17,074	16,952	16,782	16,752	16,679	16,453	16,056	15,338	15,198	-0.91%
Sweden	14,645	14,647	14,615	14,651	14,692	15,079	15,349	15,341	15,385	15,340	15,350	15,385	15,325	15,329	15,383	0.36%
United Kingdom	11,643	11,714	11,798	12,859	12,875	12,876	12,540	11,772	10,708	10,241	9,777	9,818	9,856	9,729	9,605	-1.28%

Table S15: Provincial roads network in the EU25 (Eurostat, 2011 in white; UNECE, 2011 in yellow). Orange cells are those years which were linearily inter/extrapolated (see method discussion above for details)

[km]	1995	1996	1997	1998	1999	2000	2001	2002	2003	2004	2005	2006	2007	2008	2009	Average rate of change from 1995-2009
Austria	23,472	23,472	23,472	23,472	23,065	23,086	23,236	23,386	23,535	23,685	23,685	23,644	23,687	23,652	23,673	0.06%
Belgium	1,326	1,326	1,326	1,326	1,349	1,349	1,349	1,349	1,349	1,349	1,349	1,349	1,349	1,349	1,349	0.12%
Cyprus	2,456	2,448	2,493	2,502	2,520	2,532	2,538	2,553	2,571	2,641	2,650	2,702	2,711	2,745	2,745	0.80%
Czech Republic					48,808	48,808	48,808	48,808	48,808	48,797	48,792	48,778	48,736	48,753	48,791	0.00%
Denmark	7,050	7,043	9,941	9,949	9,955	9,986	9,988	9,952	9,682	9,690	9,690	9,701	9,677	9,652	9,628	-0.25%
Estonia	28,833	28,800	27,754	33,050	34,006	34,977	35,603	36,441	37,188	37,209	38,052	38,077	38,489	38,777	39,178	1.03%
Finland					13,480	13,480	13,480	13,480	13,480	13,480	13,480	13,475	13,471	13,466	13,464	-0.01%
France	360,000	358,900	358,380	358,580	359,090	359,055	359,231	359,597	359,644	359,955	365,000	377,205	377,377	377,984	377,986	0.35%
Germany	175,970	178,343	178,346	177,852	177,899	177,780	177,834	178,298	178,237	178,324	178,134	178,117	178,172	178,151	178,269	0.09%
Greece	30,864	30,864	30,864	30,864	30,864	30,864	30,864	30,864	30,864	30,864	30,864	30,864	30,864	30,864	30,864	0.00%
Hungary					53,565	53,565	53,565	53,565	53,749	53,525	53,300	53,256	53,212	53,169	53,125	-0.08%
Ireland	10,600	10,700	11,690	11,690	11,628	11,628	11,628	11,690	11,607	11,645	11,645	11,638	11,631	11,631	11,631	0.69%
Italy	114,442	113,924	113,790	115,125	115,222	114,691	115,180	143,468	149,106	151,570	147,364	147,364	156,258	157,785	159,162	0.87%
Latvia	30,804	31,274	31,619	32,365	32,481	33,042	33,109	32,873	31,787	31,475	31,577	31,432	31,379	31,100	31,382	0.14%
Lithuania	36,666	40,238	43,240	45,987	46,828	48,451	49,475	55,813	57,560	57,986	58,169	58,659	59,394	59,710	60,062	2.39%
Luxembourg	1,897	1,907	1,907	1,911	1,911	1,891	1,891	1,891	1,891	1,891	1,891	1,891	1,891	1,891	1,910	0.05%
Malta			1,167	1,167	1,252	1,337	1,422	1,439	1,501	1,566	1,634	1,705	1,778	1,855	1,935	4.33%
Netherlands	6,910	6,314	7,192	7,365	7,539	7,712	7,885	7,866	7,856	7,799	6,882	6,878	6,944	6,834	6,827	-0.10%
Poland	128,624	128,684	128,548	128,544	156,258	156,669	156,005	156,784	157,044	157,314	156,805	156,225	155,814	155,460	154,956	-0.07%
Portugal	58,990	58,990		4,805	4,528	4,499	4,500	4,500	4,500	4,500	4,500	4,464	4,428	4,393	4,358	-0.80%
Slovakia	3,878	3,921	3,771	3,773	3,826	3,826	3,828	3,829	3,729	3,729	3,734	3,742	3,742	3,686	3,644	-0.43%
Slovenia	9,791	9,781	9,804		4,853	4,853	4,853	4,853	4,853	4,853	4,853	4,930	5,007	5,084	5,106	0.44%
Spain	71,377	70,905	71,095	69,373	69,521	69,259	69,167	67,969	68,644	68,826	68,744	68,974	68,576	68,292	68,063	-0.34%
Sweden	83,263	83,368	83,447	83,442	83,357	83,094	82,892	82,915	82,882	82,990	82,984	82,932	83,131	83,138	83,128	-0.01%
United Kingdom	34,660	34,478	34,558	35,891	36,049	36,081	36,456	37,169	38,207	38,692	38,974	39,215	39,233	39,643	40,057	1.05%

Table S16: Communal roads network in the EU25 (ERF, 2007 in grey; ERF et al., 2010 in green; Eurostat, 2011 in white; UNECE, 2011 in yellow). Orange cells are those years which were linearily inter/extrapolated (see method discussion above for details). Data for Germany is modified in comparison to statistical database due to under-representation of communal roads ((Reichel, 2012) and method discussion above for details).

[km]	1995	1996	1997	1998	1999	2000	2001	2002	2003	2004	2005	2006	2007	2008	2009	Average rate of change from 1995-2009
Austria	71,000	71,000	71,000	71,000	71,053	71,059	71,071	71,082	71,065	71,082	71,059	71,059	71,059	71,059	71,112	0.02%
Belgium	127,600	128,200	129,400	130,300	130,900	131,520	132,540	133,330	134,130	134,940	135,745	136,559	137,379	137,870	138,000	0.56%
Cyprus	2,839	3,015	3,103	3,155	3,221	3,271	3,352	3,404	3,481	3,577	3,666	3,732	3,946	3,975	4,019	2.53%
Czech Republic	66,449	70,520	72,300	72,300	72,300	72,300	72,300	72,300	72,927	72,927	72,927	74,919	74,919	74,919	74,919	0.87%
Denmark	59,711	59,710	59,861	59,882	59,995	60,018	60,240	60,328	60,717	60,894	61,006	61,024	69,331	69,492	69,632	0.20%
Estonia						38,284	38,685	39,091	39,500	39,909	40,323	40,741	41,163	41,547	41,978	1.04%
Finland	22,000	22,000	23,593	24,051	24,262	24,448	24,805	24,828	25,198	25,697	26,213	26,702	27,442	27,365	28,318	1.84%
France	563,000	574,780	579,410	585,900	589,910	594,149	601,733	598,380	601,851	606,031	610,330	615,607	628,987	629,000	642,256	0.95%
Germany	519,188	519,253	521,554	521,527	521,054	521,076	520,977	520,996	521,430	521,427	521,588	521,581	521,520	521,565	521,623	0.19%
Greece						75,600	75,600	75,600	75,600	75,600	75,600	75,600	75,600	75,600	75,600	0.00%
Hungary				75,936	75,936	75,936	75,936	75,936	75,930	75,930	75,930	75,930	75,930	75,930	75,930	0.00%
Ireland	76,500	76,600	78,610	78,610	78,675	78,675	78,675	78,610	78,773	79,446	79,446	79,202	78,958	78,958	78,958	0.32%
Italy	660,566	663,503	668,669	660,000	660,000	660,000	660,000	660,000	660,000	660,000	660,000	616,510	648,470	654,940	659,385	0.68%
Latvia	4,990	5,074	5,039	5,124	5,162	5,201	7,210	7,320	7,338	7,345	7,575	7,581	7,796	7,949	7,872	1.01%
Lithuania	4,726	4,853	4,882	5,308	5,551	5,753	5,783	5,983	6,190	6,404	6,625	6,855	7,092	7,337	7,591	3.46%
Luxembourg																0.00%
Malta			647	647	647	647	647	647	647	647	647	647	647	647	647	0.00%
Netherlands	103,304	106,095	105,680	108,677	111,675	114,672	117,669	118,667	119,437	120,447	118,328	118,979	119,489	120,100	120,685	0.49%
Poland	198,178	200,631	202,852	206,825	197,351	198,350	203,505	197,389	201,992	203,773	206,371	207,951	208,693	209,333	210,569	0.45%
Portugal					63,900	63,900	63,900	63,900	63,900	63,900	63,900	64,330	64,763	65,199	65,638	0.27%
Slovakia	10,718	10,658	10,418	10,427	10,393	10,394	10,391	10,396	10,396	10,394	36,343	36,341	36,344	36,344	36,348	-0.23%
Slovenia				31,198	32,006	32,131	32,242	32,171	32,059	32,102	32,092	32,138	32,233	32,219	32,224	0.30%
Spain	66,984	66,634	66,781	68,499	68,160	68,126	67,987	69,479	68,862	68,854	68,791	68,839	68,366	67,948	68,026	0.12%
Sweden	38,900	38,500	38,500	38,500	39,523	39,500	40,000	40,280	40,300	40,300	40,300	41,000	41,000	41,000	41,168	0.41%
United Kingdom	336,168	337,494	338,158	360,918	361,651	362,360	363,104	363,918	364,659	359,991	360,563	366,732	367,317	344,217	344,962	0.22%

Table S17: Railway network of single track lines in the EU25 (Eurostat 2011 in white; UNECE 2011 in yellow) . Orange cells are those years which were linearily inter/extrapolated (see method discussion above for details)

[km]	1995	1996	1997	1998	1999	2000	2001	2002	2003	2004	2005	2006	2007	2008	2009	Rate of change
Austria	3,905	3,905	3,905	3,876	3,973	3,999	4,024	4,050	4,076	4,101	4,127	4,152	4,178	4,202	4,226	0.57%
Belgium	870	806	800	827	771	766	753	784	816	824	820	825	828	825	796	-0.35%
Cyprus						0	0	0	0	0	0	0	0	0	0	0.00%
Czech Republic	7,410	7,494	7,490	7,490	7,515	7,515	7,645	7,725	7,757	7,746	7,746	7,746	7,719	7,679	7,684	0.28%
Denmark					2,396	2,387	2,387	2,372	2,372	2,235	2,235	2,197	2,235	2,235	2,218	-0.75%
Estonia	917	916	862	862	863	863	852	863	851	930	1,010	1,089	1,093	1,089	1,089	1.42%
Finland	5,363	5,364	5,358	5,360	5,329	5,347	5,343	5,343	5,344	5,234	5,225	5,335	5,329	5,349	5,349	-0.02%
France	15,950	15,852	15,862	15,831	15,794	15,460	15,243	15,178	14,856	14,745	14,629	14,604	14,578	14,383	12,966	-0.74%
Germany	24,087	23,072	21,009	20,696	19,970	18,878	18,241	17,920	17,636	18,773	19,909	19,554	19,205	18,862	18,526	-1.78%
Greece	2,180	2,170	2,199	1,978	1,978	2,031	2,021	2,027	2,006	2,018	2,059	2,021	2,042	2,042	2,049	0.35%
Hungary	6,437	6,416	6,380	6,349	6,359	6,375	6,387	6,384	6,389	6,392	6,393	6,962	6,639	6,639	6,055	0.30%
Ireland	1,467	1,515	1,418	1,419	1,419	1,419	1,419	1,414	1,409	1,404	1,399	1,298	1,196	1,340	1,318	-1.63%
Italy	9,982	9,969	9,924	9,945	9,889	9,818	9,805	9,720	9,667	9,554	9,451	9,397	9,285	9,223	9,492	-0.61%
Latvia	2,109	2,109	2,110	2,110	2,110	2,026	2,002	1,967	1,967	1,967	1,967	1,966	1,962	1,960	1,571	-0.56%
Lithuania	1,442	1,455	1,432	1,432	1,340	1,339	1,165	1,344	1,370	1,397	1,387	1,387	1,384	1,381	1,385	-0.40%
Luxembourg	135	134	134	134	134	134	134	134	135	135	135	135	135	135	135	0.00%
Malta						0	0	0	0	0	0	0	0	0	0	0.00%
Netherlands	978	960	952	931	931	925	931	930	924	924	918	902	904	906	901	-0.58%
Poland	15,081	14,536	14,532	14,337	13,993	13,799	12,335	12,117	11,769	11,458	11,506	11,429	11,052	11,458	11,621	-1.33%
Portugal	2,609	2,600	2,566	2,329	2,317	2,317	2,317	2,281	2,273	2,266	2,258	2,250	2,242	2,234.5	2,235	-0.62%
Slovakia	2,637	2,657	2,657	2,645	2,645	2,642	2,642	2,637	2,637	2,640	2,639	2,640	2,610	2,607	2,608	-0.09%
Slovenia	869	869	869	870	871	871	898	898	898	898	898	898	898	898	898	0.25%
Spain	8,998	8,940	8,898	8,898	8,905	8,946	8,946	8,923	8,925	8,927	8,909	8,881	8,811	8,748	8,736	-0.22%
Sweden	9,476	9,498	9,431	9,462	9,469	9,328	9,302	9,356	9,270	9,258	9,233	9,217	9,166	9,197	9,297	-0.23%
United Kingdom	10,958	10,958	10,945	10,955	11,404	11,745	11,794	11,770	11,785	11,769	11,776	11,773	11,780	11,779	11,846	0.57%

Table S18: Railway network for double or more tracks in the EU25 (Eurostat 2011 in white; UNECE 2011 in yellow). Orange cells are those years which were linearily inter/extrapolated (see method discussion above for details)

GEO/TIME	1995	1996	1997	1998	1999	2000	2001	2002	2003	2004	2005	2006	2007	2008	2009	
Austria	1,767	1,767	1,767	1,767	1,767	1,806	1,845	1,884	1,923	1,961	2,000	2,039	2,078	2,106	2,135	1.36%
Belgium	2,558	2,574	2,622	2,643	2,701	2,705	2,701	2,934	2,705	2,712	2,724	2,735	2,740	2,769	2,782	0.60%
Cyprus																0.00%
Czech Republic	1,917	1,941	1,940	1,940	1,929	1,929	1,878	1,875	1,845	1,866	1,868	1,851	1,869	1,907	1,894	-0.08%
Denmark			877	893	893	914	914	940	946	942	942	942	946	946	953	0.70%
Estonia	104	104	104	105	105	105	115	101	108	108	107	107	107	107	107	0.58%
Finland	496	496	507	507	507	507	507	507	507	507	507	570	570	570	570	0.17%
France	15,989	16,000	15,958	15,939	15,941	15,937	16,142	16,142	16,133	16,135	16,242	16,409	16,576	16,658	16,500	0.23%
Germany	17,631	17,754	17,376	17,430	17,555	17,709	17,745	17,884	17,957	18,127	18,297	18,297	18,359	18,422	18,485	0.34%
Greece	294	304	304	321	321	354	356	356	408	431	517	488	509	510	517	1.41%
Hungary	1,195	1,203	1,213	1,293	1,292	1,293	1,292	1,292	1,292	1,292	1,292	1,173	1,173	1,173	1,335	-0.09%
Ireland	478	439	490	490	500	500	500	503	507	510	513	575	638	460	458	-0.39%
Italy	6,023	6,045	6,106	6,135	6,203	6,156	6,230	6,265	6,297	6,362	6,774	6,898	7,050	7,306	7,493	1.59%
Latvia	304	304	303	303	303	305	303	303	303	303	303	303	303	303	313	0.21%
Lithuania	560	542	565	565	565	566	531	431	404	385	385	385	383	382	383	-1.27%
Luxembourg	140	140	140	140	140	140	140	140	140	140	140	140	140	140	140	0.00%
Malta	0	0	0	0	0	0	0	0	0	0	0	0	0	0	0	0.00%
Netherlands	1,835	1,853	1,853	1,877	1,877	1,877	1,878	1,876	1,877	1,877	1,892	1,894	1,897	1,982	1,994	0.60%
Poland	8,905	8,884	8,892	8,873	8,898	8,761	8,784	8,956	8,896	8,792	8,747	8,747	8,745	8,738	8,739	-0.13%
Portugal	455	471	472	465	497	497	497	520	532	545	557	570	582	595	607	2.15%
Slovakia	1,028	1,016	1,016	1,020	1,020	1,020	1,020	1,020	1,020	1,020	1,019	1,018	1,019	1,016	1,015	-0.09%
Slovenia	332	332	332	331	330	330	330	331	331	331	330	330	330	330	333	0.03%
Spain	3,282	3,344	3,405	3,405	3,414	3,364	3,364	3,375	3,643	3,910	3,930	4,127	4,557	4,605	4,618	2.53%
Sweden	1,449	1,466	1,510	1,535	1,575	1,709	1,719	1,740	1,768	1,793	1,785	1,804	1,807	1,826	1,842	1.75%
United Kingdom	4,856	4,856	4,850	4,855	5,054	5,205	5,226	5,216	5,223	5,215	5,218	5,218	5,221	5,220	5,249	0.57%
